# Supplementary material for: Racemic Norlignans as Diastereoisomers from Ferula sinkiangensis Resins with Antitumor and Wound-Healing Promotion Activities
Source: Molecules. 2022 Jun 17;27(12):3907. doi: 10.3390/molecules27123907 (PMC9228879; doi:10.3390/molecules27123907)
Supplement: Supplementary file 1 [file molecules-27-03907-s001.zip › molecules-1746314-supplementary.pdf]

Supplementary Materials

Racemic Norlignans as Diastereoisomers from *Ferula sinkiangensis* Resins with Antitumor and Wound-Healing Promotion Activities

Ying-Shi Li [1,2,†], Bao-Chen Yang [2,†], Shu-Min Zheng [2], Yong-Xian Cheng [2,3,*] and Hong-Hua Cui [1,*]

[1] School of Traditional Chinese Medicine, Guangdong Pharmaceutical University, Guangzhou 510006, China; lws1030@163.com

[2] Institute for Inheritance-Based Innovation of Chinese Medicine, School of Pharmaceutical Sciences, Health Science Center, Shenzhen University, Shenzhen 518060, China; ybc15801655293@hotmail.com (B.-C.Y.); zheng80231223@163.com (S.-M.Z.)

[3] Guangdong Key Laboratory of Functional Substances in Medicinal Edible Resources and Healthcare Products, School of Life Sciences and Food Engineering, Hanshan Normal University, Chaozhou 521041, China

* Correspondence: yxcheng@szu.edu.cn (Y.-X.C.); honghuacui@gdpu.edu.cn (H.-H.C.)

† These authors contributed equally to this work.

1. Supplementary Figures

Figure S1. ^{1}H NMR (600 MHz) spectrum of **1** in methanol-d_4
Figure S2. ^{13}C NMR and DEPT (150 MHz) spectra of **1** in methanol-d_4
Figure S3. ^{1}H-^{1}H COSY (600 MHz) spectrum of **1** in methanol-d_4
Figure S4. HSQC (600 MHz) spectrum of **1** in methanol-d_4
Figure S5. HMBC (600 MHz) spectrum of **1** in methanol-d_4
Figure S6. ROESY (600 MHz) spectrum of **1** in methanol-d_4
Figure S7. HR-ESI-MS spectrum of **1**
Figure S8. UV spectrum of **1**
Figure S9. The HPLC chromatogram on chiral resolution of **1** by Daicel Chiralpak IC column.
Figure S10. ^{1}H NMR (600 MHz) spectrum of **2** in methanol-d_4
Figure S11. ^{13}C NMR and DEPT (150 MHz) spectra of **2** in methanol-d_4
Figure S12. ^{1}H-^{1}H COSY (600 MHz) spectrum of **2** in methanol-d_4
Figure S13. HSQC (600 MHz) spectrum of **2** in methanol-d_4
Figure S14. HMBC (600 MHz) spectrum of **2** in methanol-d_4
Figure S15. ROESY (600 MHz) spectrum of **2** in methanol-d_4
Figure S16. HR-ESI-MS spectrum of **2**
Figure S17. UV spectrum of **2**
Figure S18. The HPLC chromatogram on chiral resolution of **2** by Daicel Chiralpak AD-H column.
Figure S19. ^{1}H NMR (600 MHz) spectrum of **3** in methanol-d_4
Figure S20. ^{13}C NMR and DEPT (150 MHz) spectra of **3** in methanol-d_4
Figure S21. ^{1}H-^{1}H COSY (600 MHz) spectrum of **3** in methanol-d_4
Figure S22. HSQC (600 MHz) spectrum of **3** in methanol-d_4
Figure S23. HMBC (600 MHz) spectrum of **3** in methanol-d_4
Figure S24. ROESY (600 MHz) spectrum of **3** in methanol-d_4
Figure S25. HR-ESI-MS spectrum of **3**
Figure S26. UV spectrum of **3**
Figure S27. The HPLC chromatogram on chiral resolution of **3** by Daicel Chiralpak IC column
Figure S28. ^{1}H NMR (600 MHz) spectrum of **4** in methanol-d_4
Figure S29. ^{13}C NMR and DEPT (150 MHz) spectra of **4** in methanol-d_4
Figure S30. ^{1}H-^{1}H COSY (600 MHz) spectrum of **4** in methanol-d_4
Figure S31. HSQC (600 MHz) spectrum of **4** in methanol-d_4

Figure S32. HMBC (600 MHz) spectrum of **4** in methanol-d_4
Figure S33. ROESY (600 MHz) spectrum of **4** in methanol-d_4
Figure S34. HR-ESI-MS spectrum of **4**
Figure S35. UV spectrum of **4**
Figure S36. The HPLC chromatogram on chiral resolution of **4** by Daicel Chiralpak AD-H column
Figure S37. B3LYP/6-31G (d, p) optimized lowest energy conformers 1–8 for **1**.
Figure S38. B3LYP/6-31G (d, p) optimized lowest energy conformers 1–8 for **2**.
Figure S39. B3LYP/6-31G (d, p) optimized lowest energy conformers 1–12 for **3**.
Figure S40. B3LYP/6-31G (d, p) optimized lowest energy conformers 1–8 for **4**.

2. Supplementary Tables

Table S1. The Cartesian coordinates of the lowest energy conformers for **1**.
Table S2. The Cartesian coordinates of the lowest energy conformers for **2**.
Table S3. The Cartesian coordinates of the lowest energy conformers for **3**.
Table S4. The Cartesian coordinates of the lowest energy conformers for **4**.

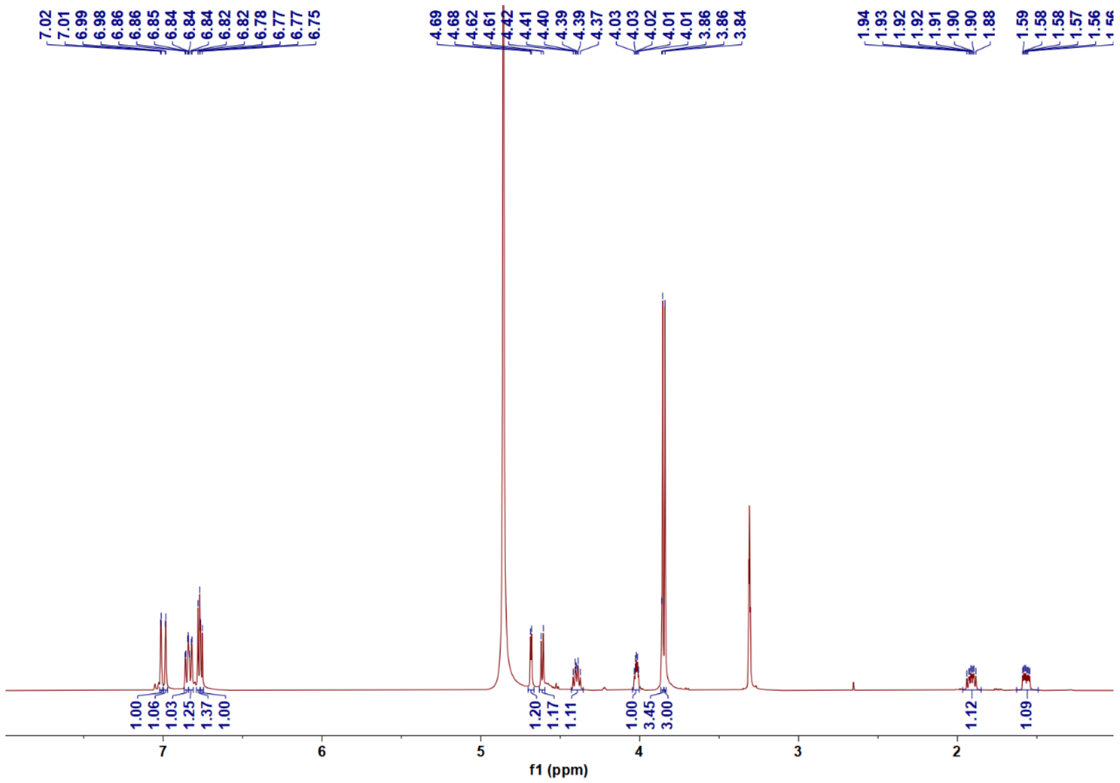

Figure S1. ^{1}H NMR (600 MHz) spectrum of **1** in methanol-d_4.

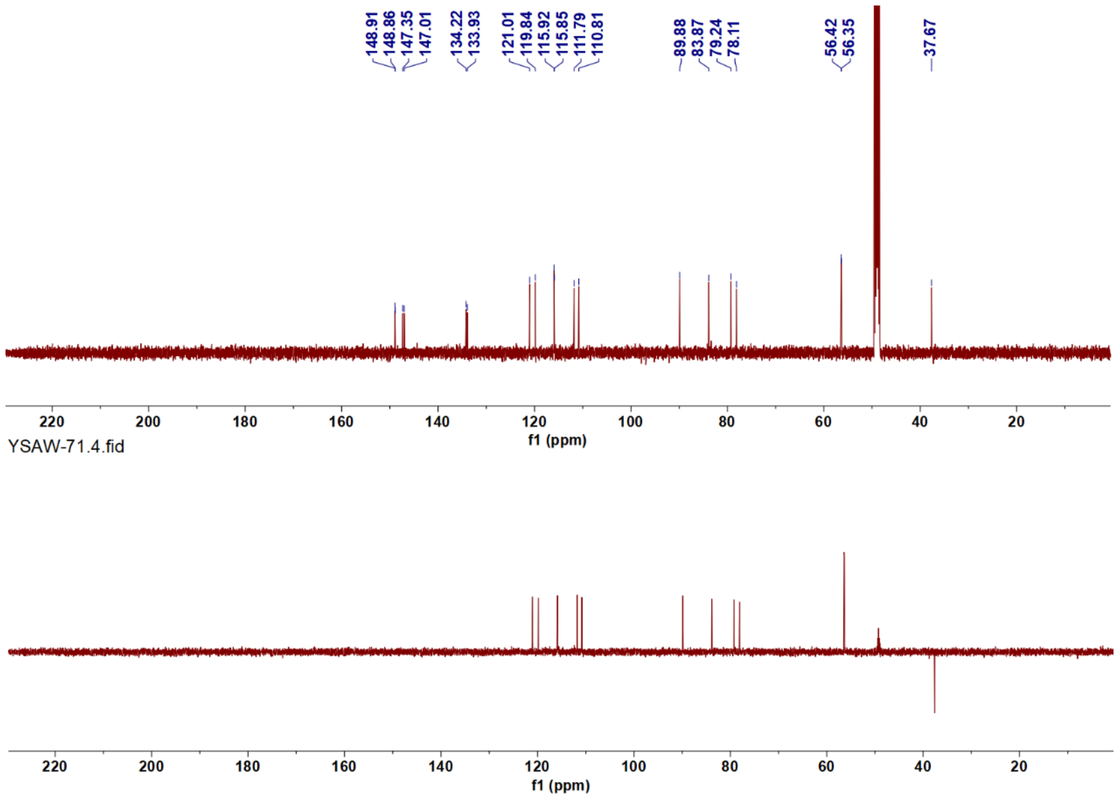

Figure S2. ^{13}C NMR and DEPT (150 MHz) spectra of **1** in methanol-d_4.

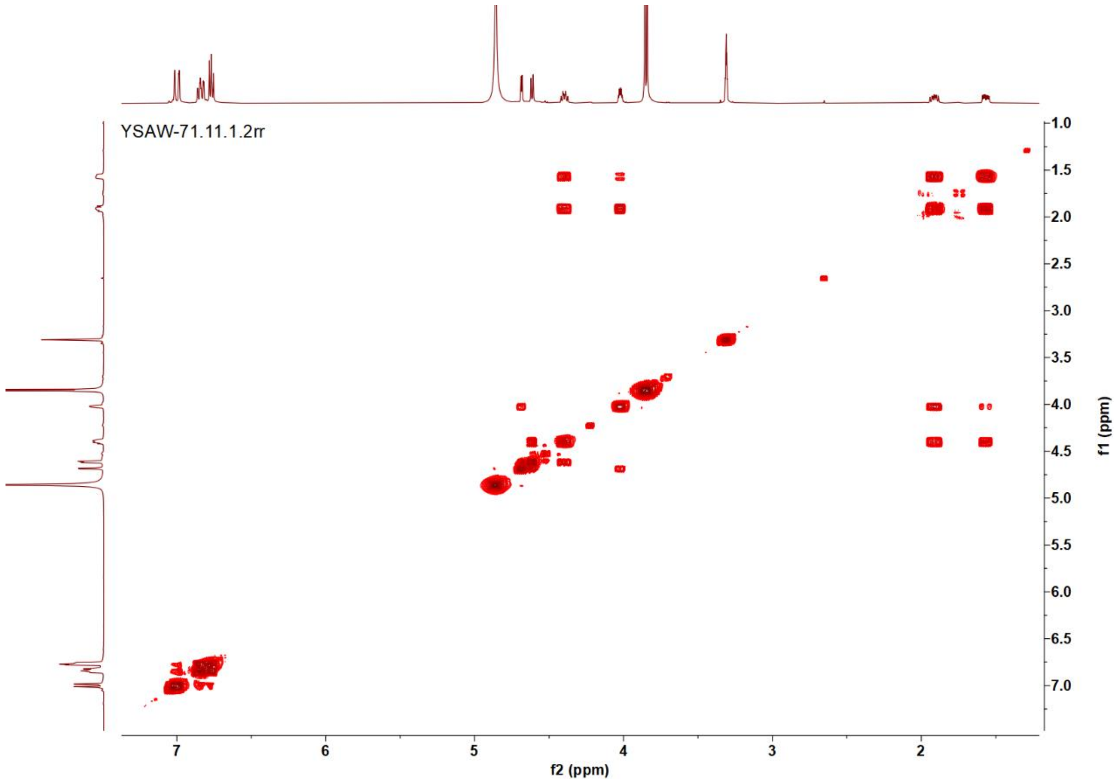

Figure S3. ^{1}H-^{1}H COSY (600 MHz) spectrum of **1** in methanol-d_4.

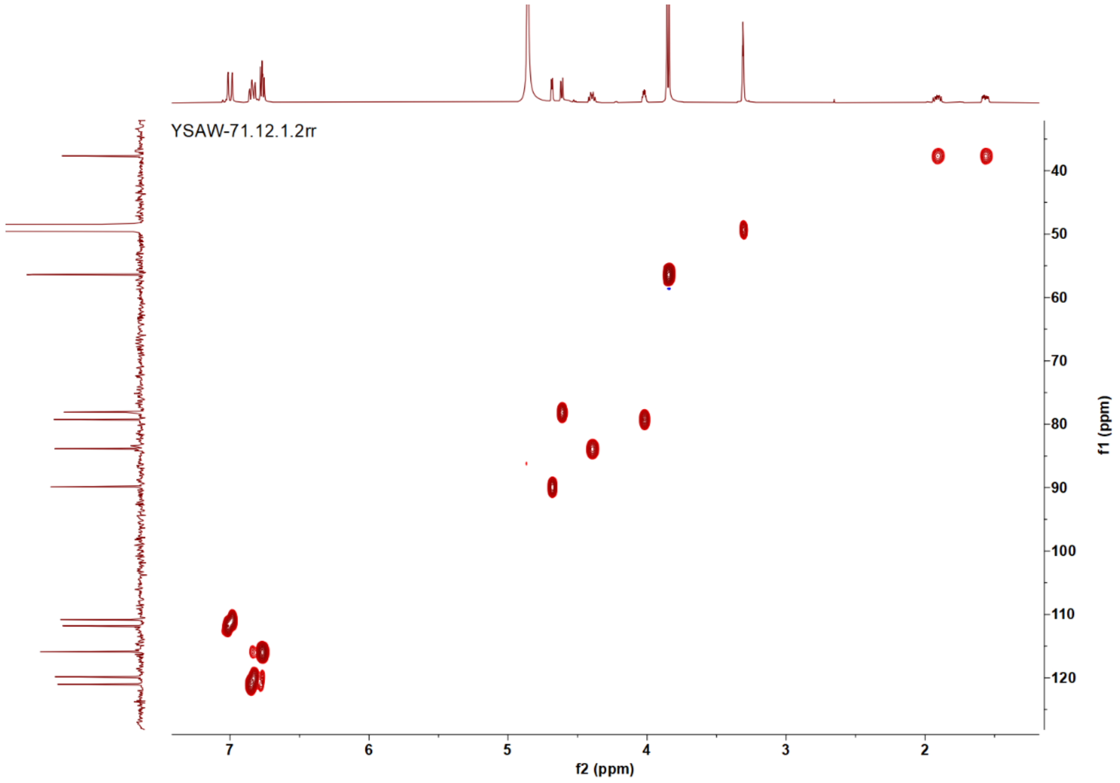

Figure S4. HSQC (600 MHz) spectrum of **1** in methanol-d_4.

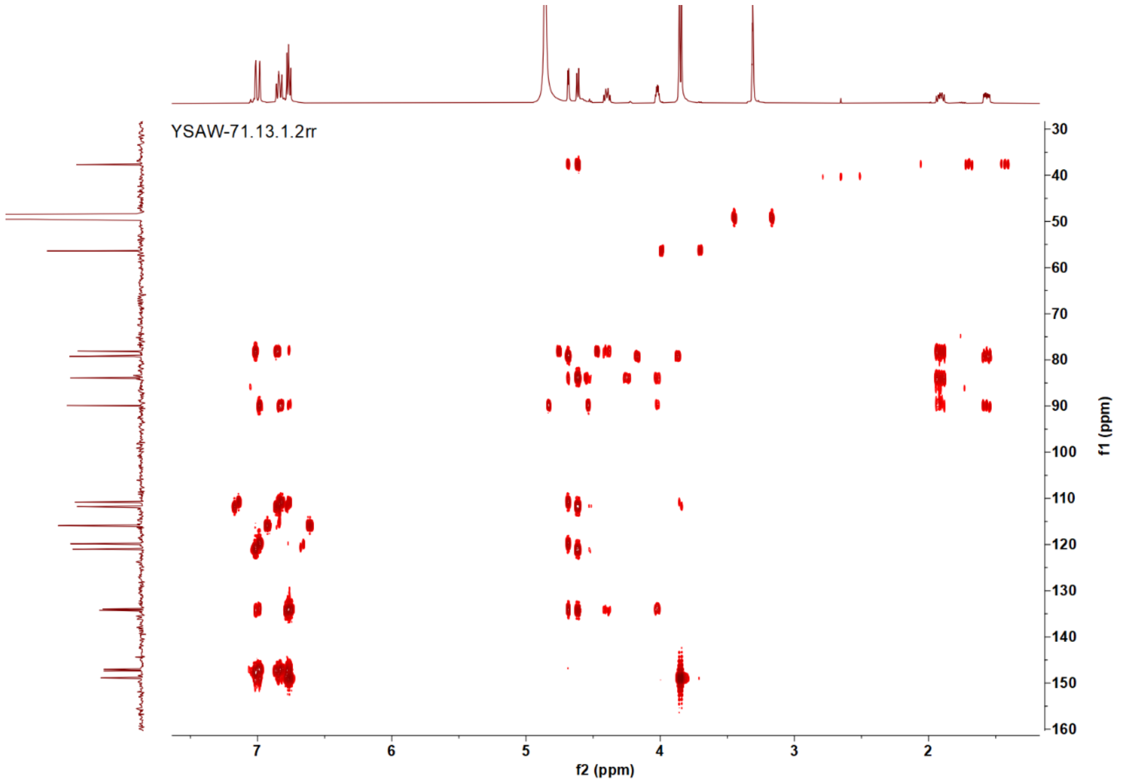

Figure S5. HMBC (600 MHz) spectrum of **1** in methanol-d_4

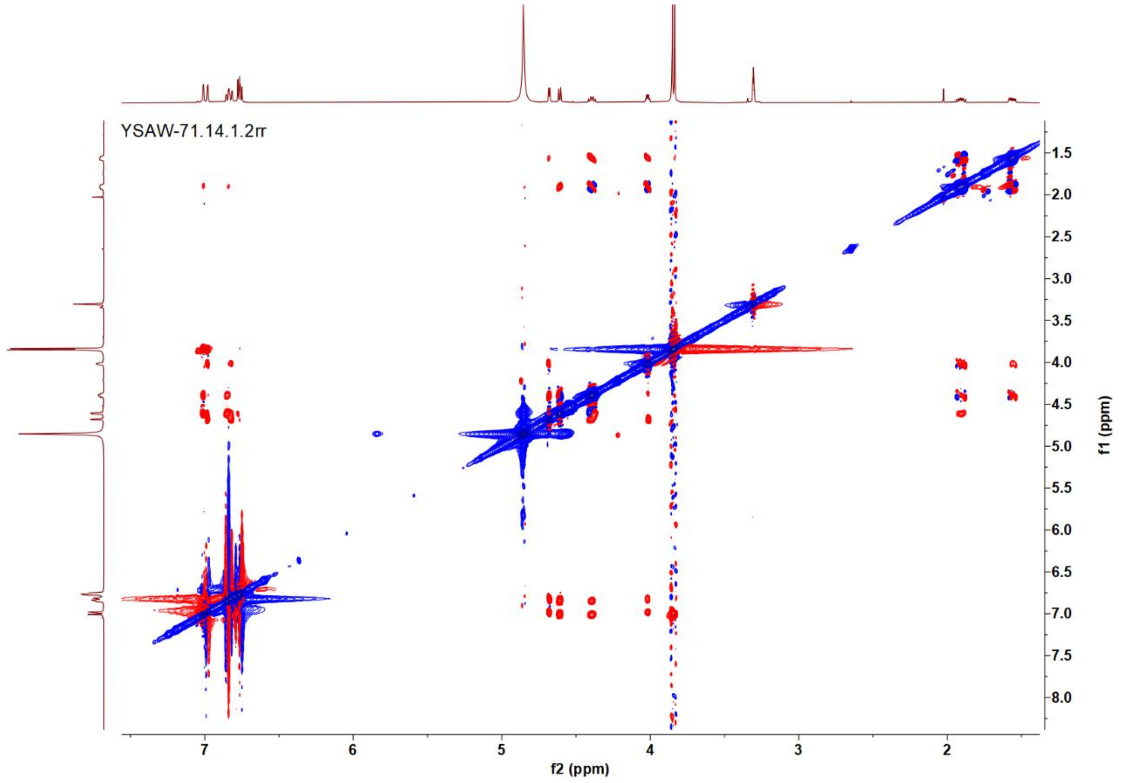

Figure S6. ROESY (600 MHz) spectrum of **1** in methanol-d_4.

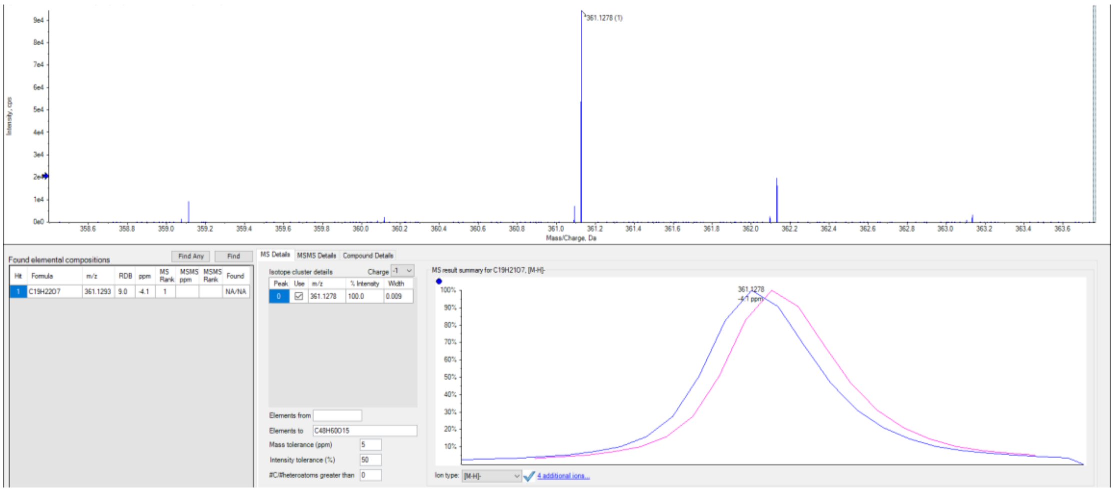

Figure S7. HR-ESI-MS spectrum of **1.**

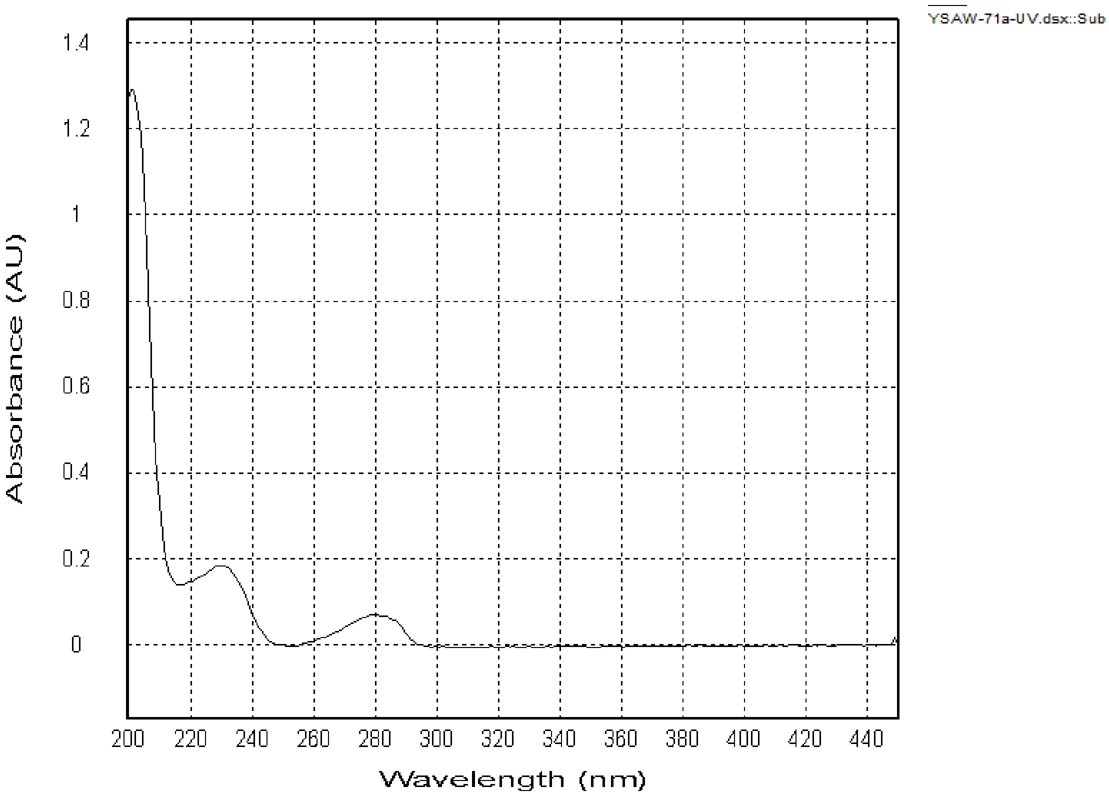

Figure S8. UV spectrum of **1.**

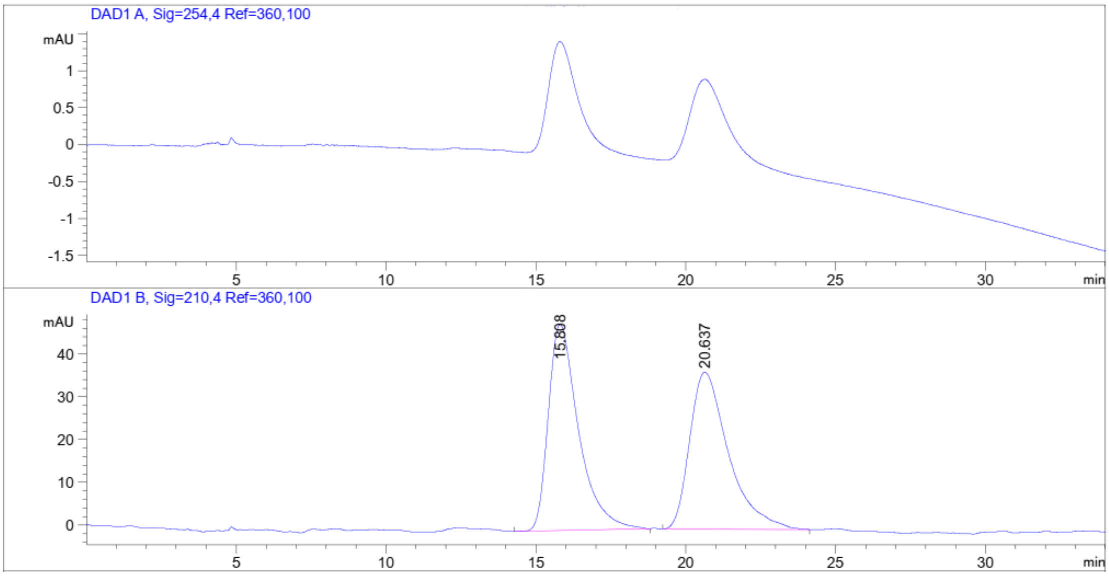

Figure S9. The HPLC chromatogram on chiral resolution of **1** by Daicel Chiralpak IC column (250 mm × 4.6 mm, i.d., 5 μm).

Analysis condition: n-hexane/iPrOH 65:35, flow rate: 1.0 mL/min.

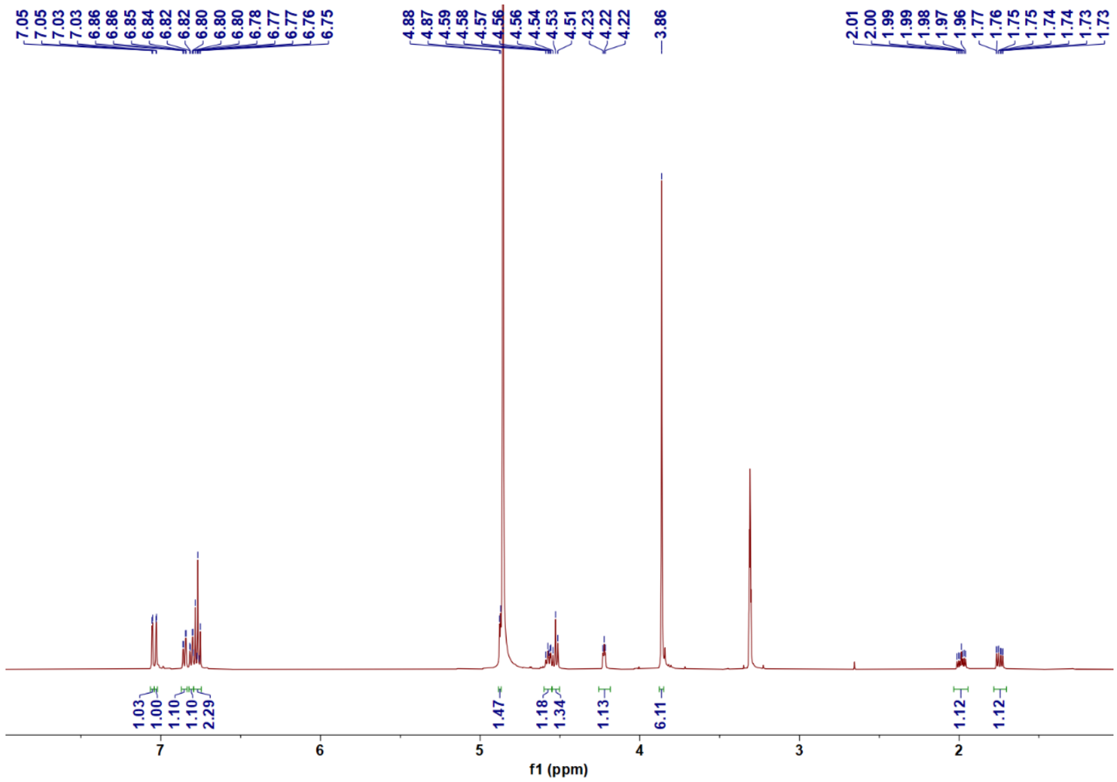

Figure S10. ^{1}H NMR (600 MHz) spectrum of **2** in methanol-d_4.

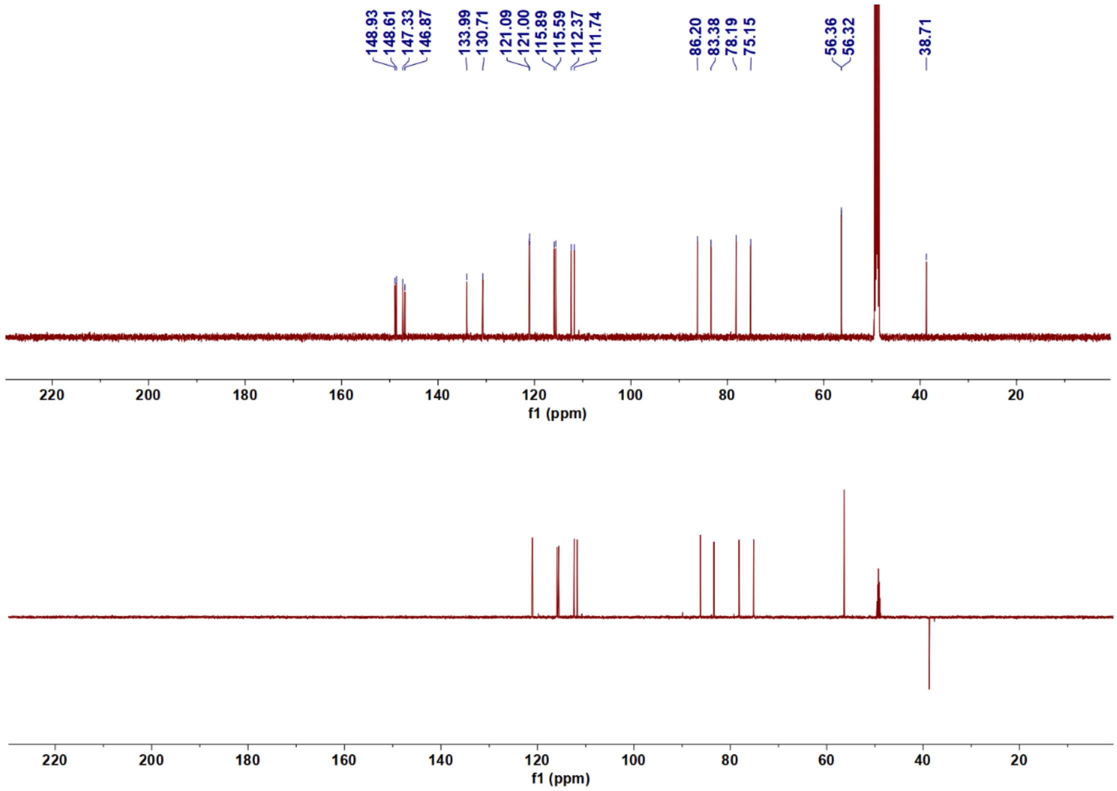

Figure S11. ^{13}C NMR and DEPT (150 MHz) spectra of **2** in methanol-d_4.

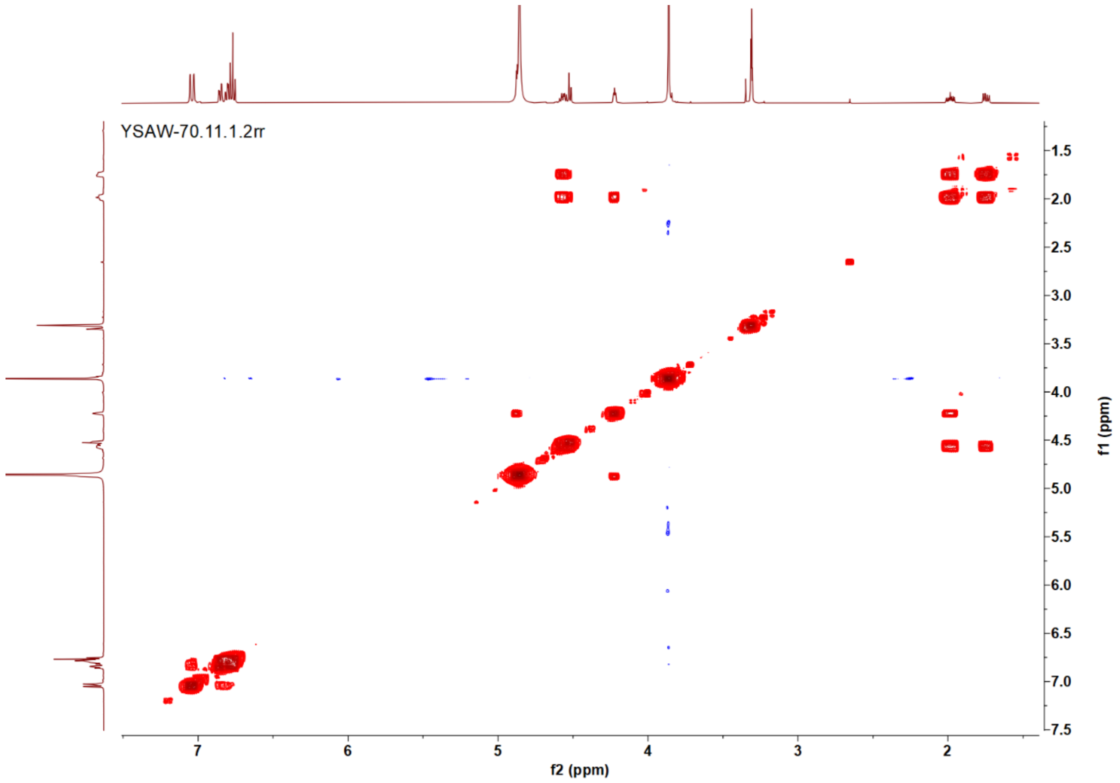

Figure S12. ^{1}H-^{1}H COSY (600 MHz) spectrum of **2** in methanol-d_4.

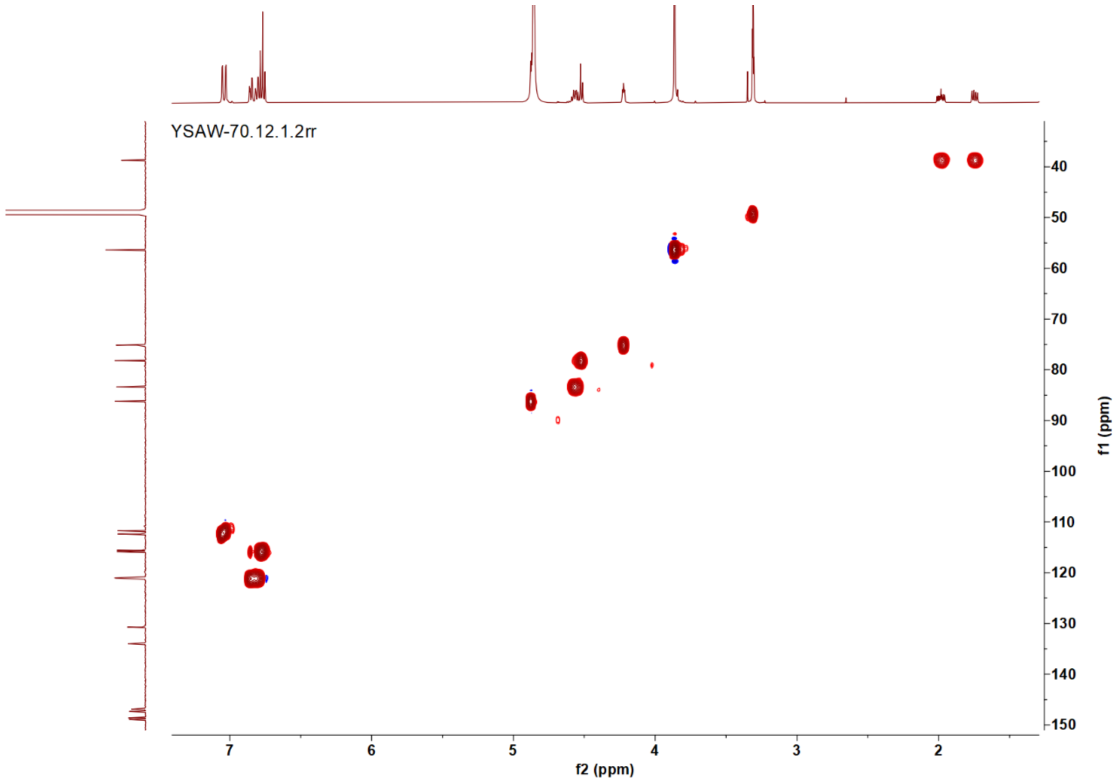

Figure S13. HSQC (600 MHz) spectrum of **2** in methanol-d_4.

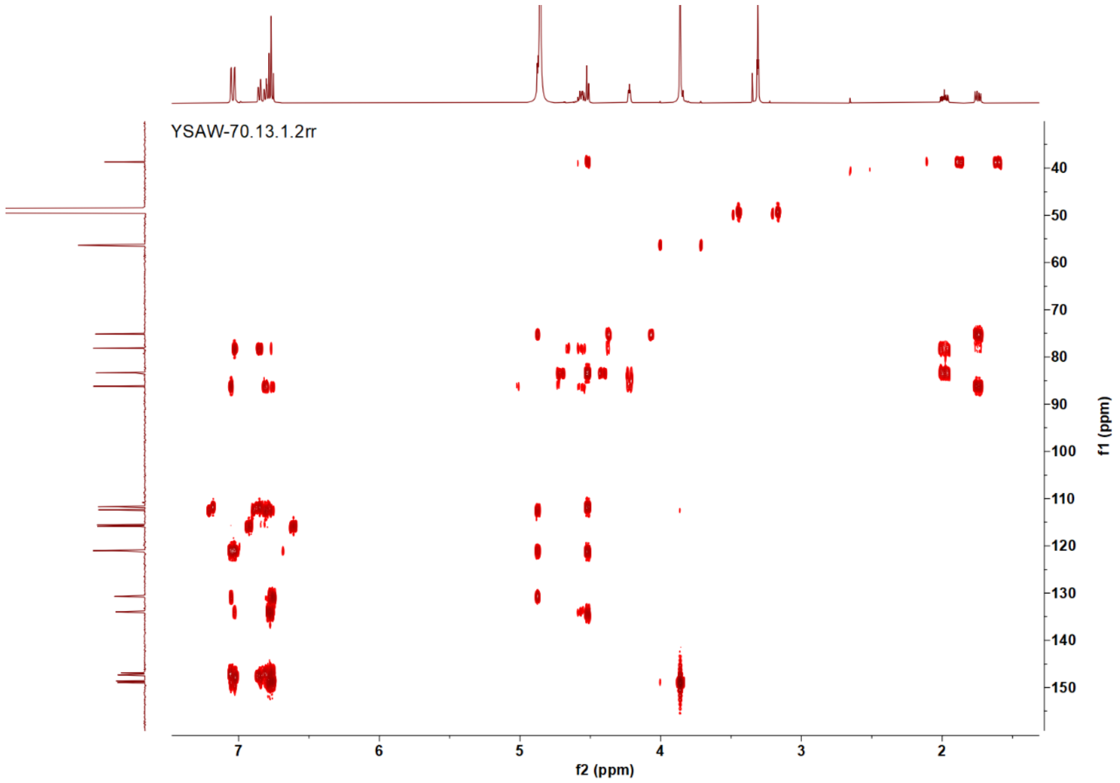

Figure S14. HMBC (600 MHz) spectrum of **2** in methanol-*d*4.

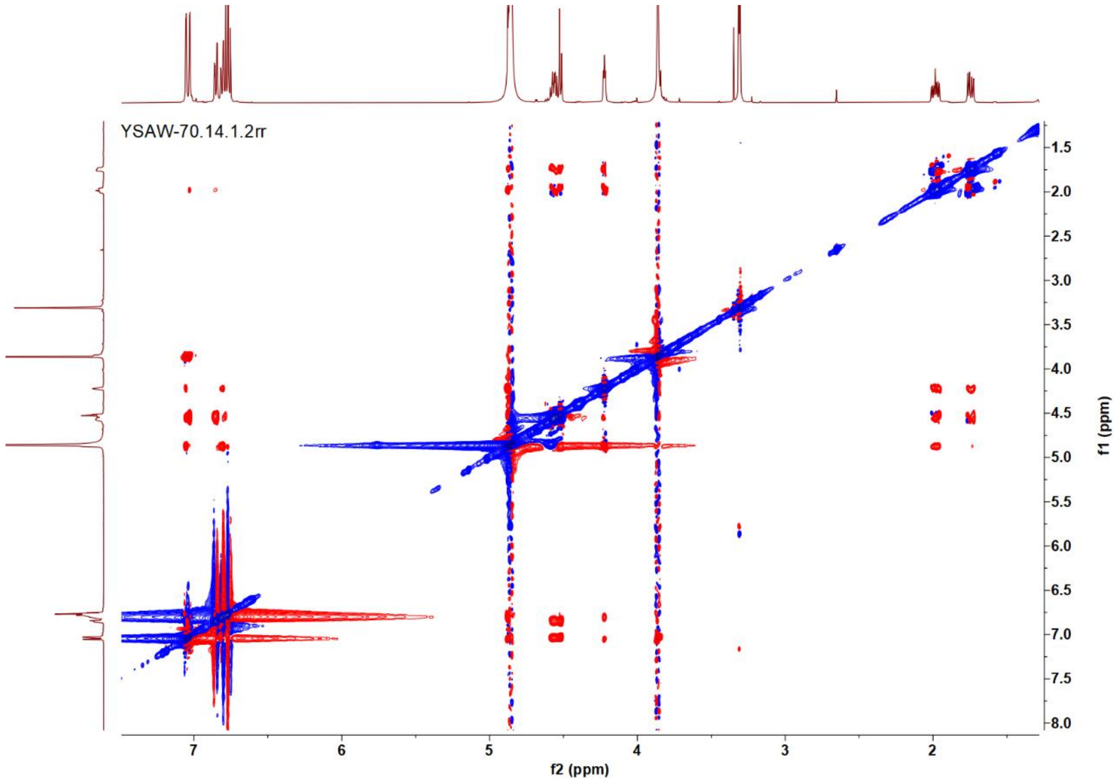

Figure S15. ROESY (600 MHz) spectrum of **2** in methanol-*d*4.

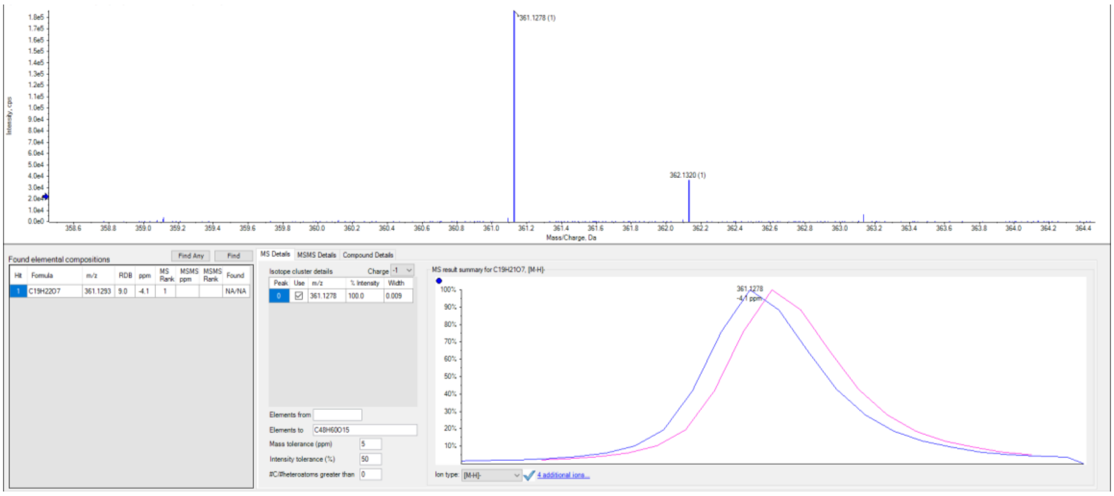

Figure S16. HR-ESI-MS spectrum of **2**.

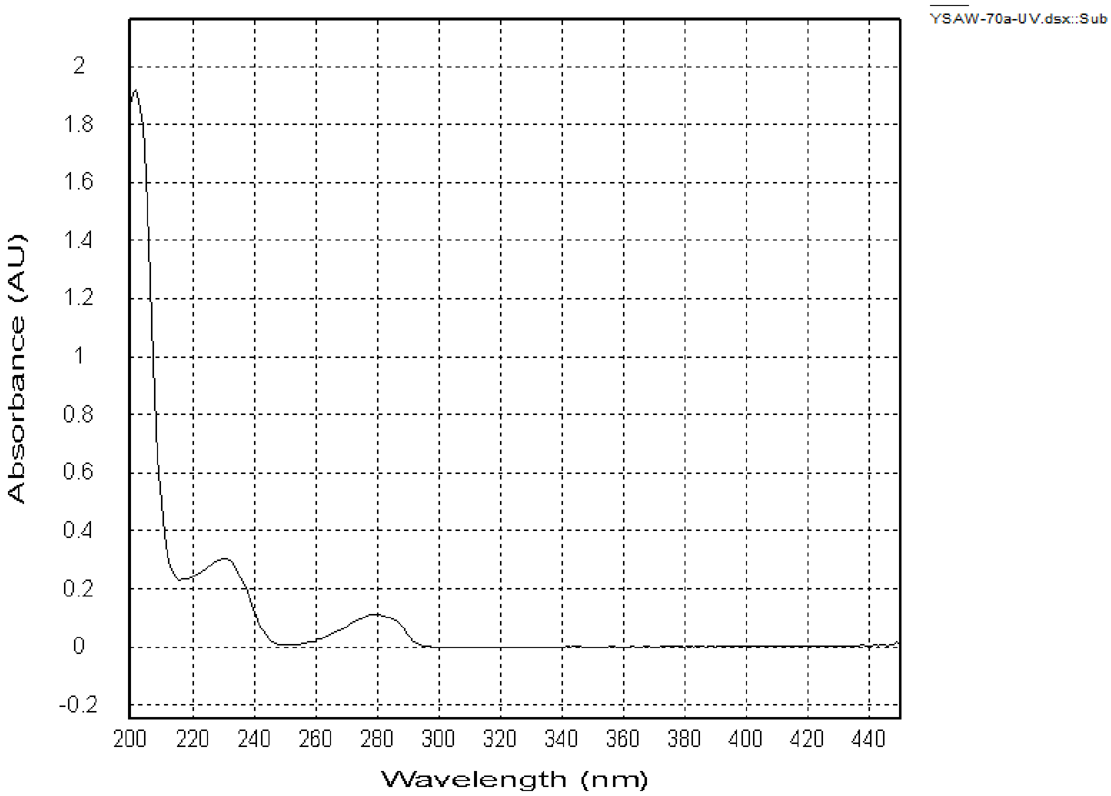

Figure S17. UV spectrum of **2.**

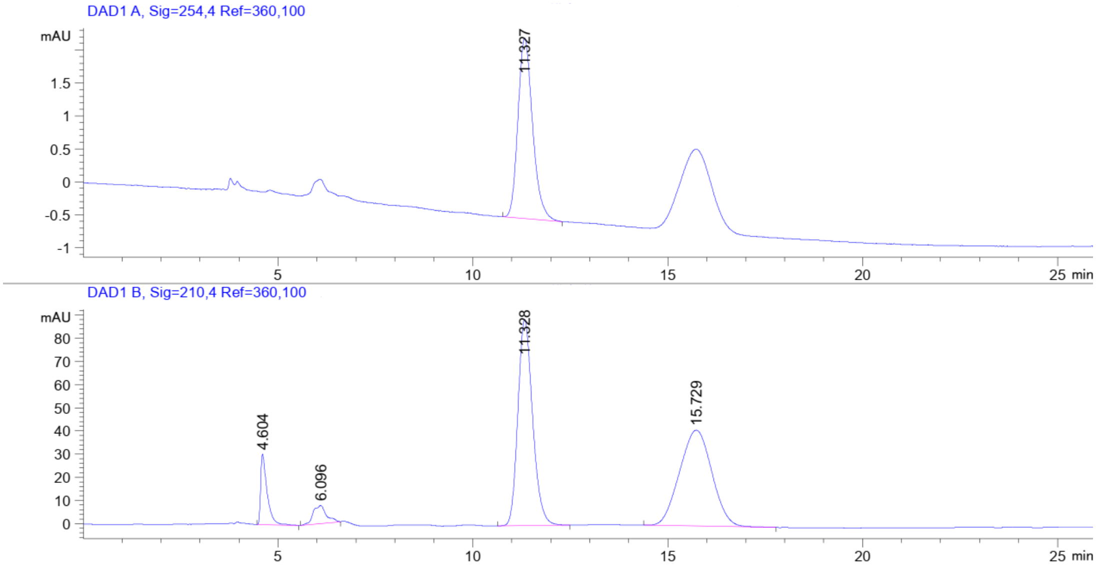

Figure S18. The HPLC chromatogram on chiral resolution of **2** by Daicel Chiralpak AD-H column (250 mm × 4.6 mm, i.d., 5 μm).

Analysis condition: n-hexane/EtOH 55:45, flow rate: 1.0 mL/min.

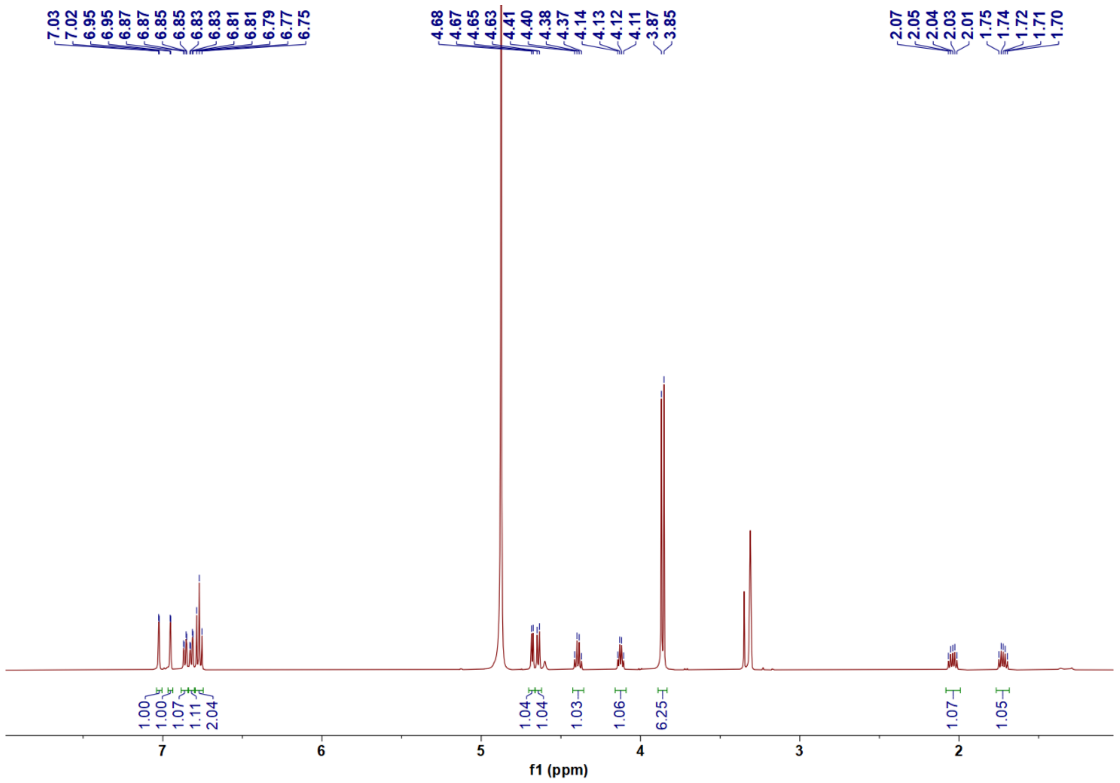

Figure S19. ^{1}H NMR (600 MHz) spectrum of **3** in methanol-*d*4.

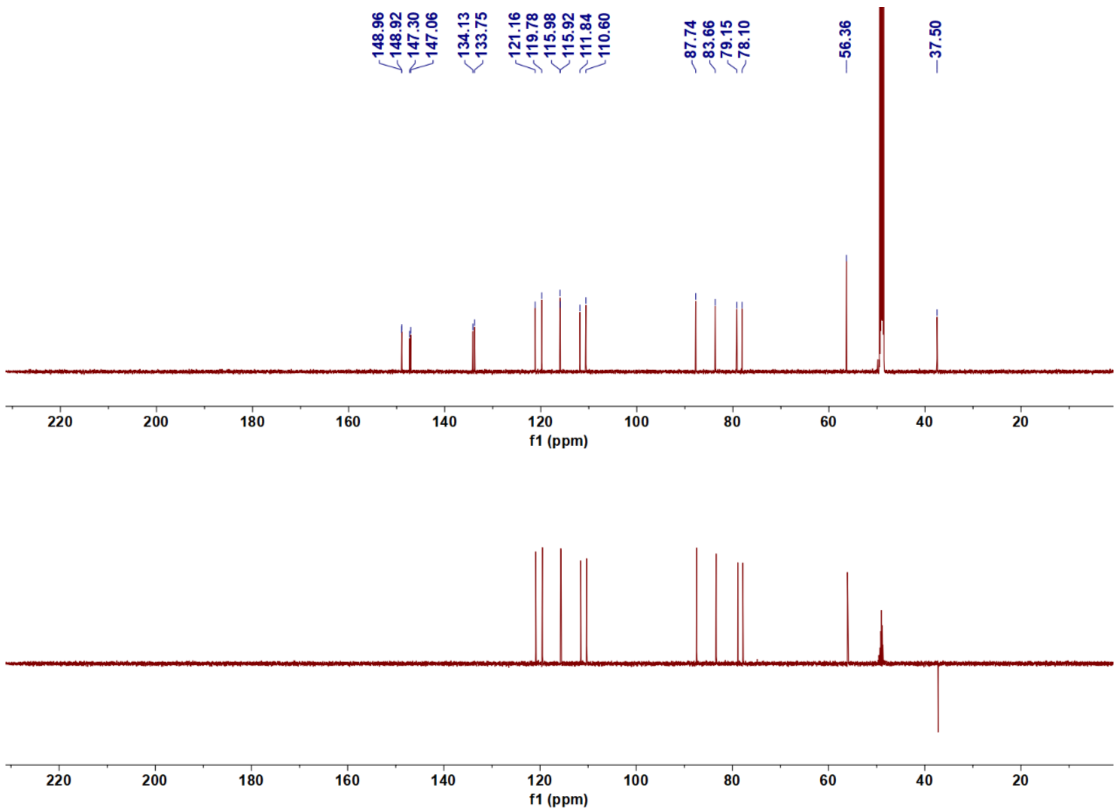

Figure S20. ^{13}C NMR and DEPT (150 MHz) spectra of **3** in methanol-*d*4.

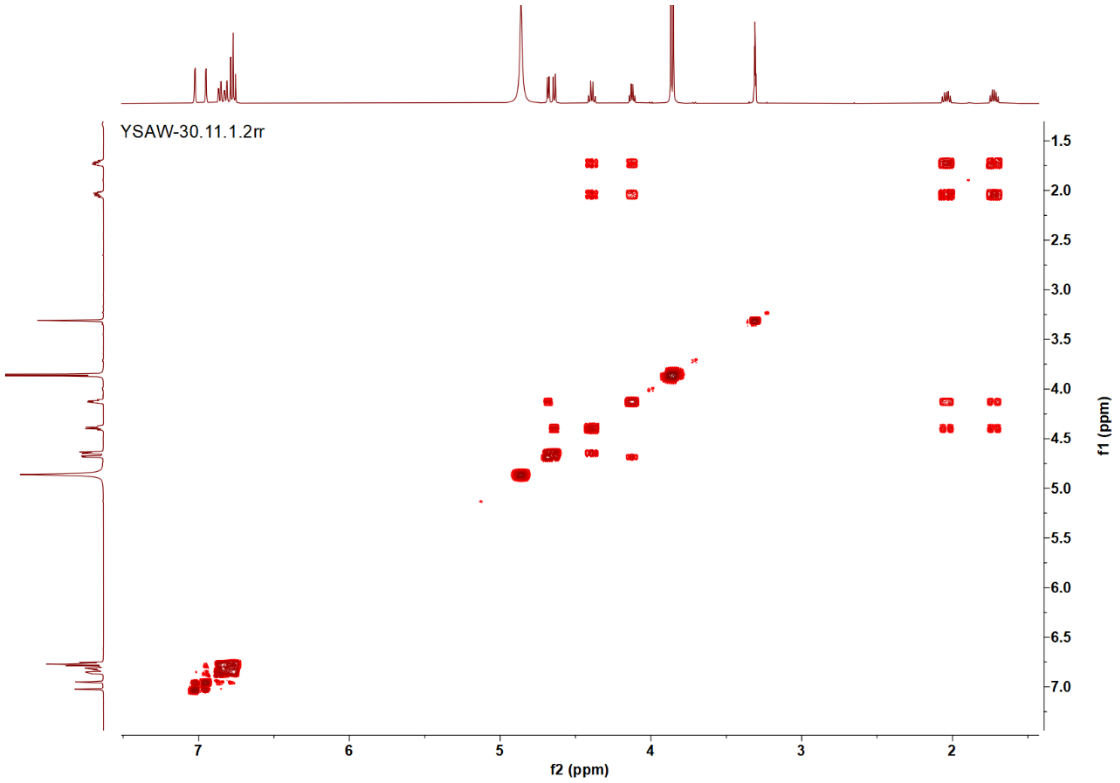

Figure S21. ^{1}H-^{1}H COSY (600 MHz) spectrum of **3** in methanol-d_4.

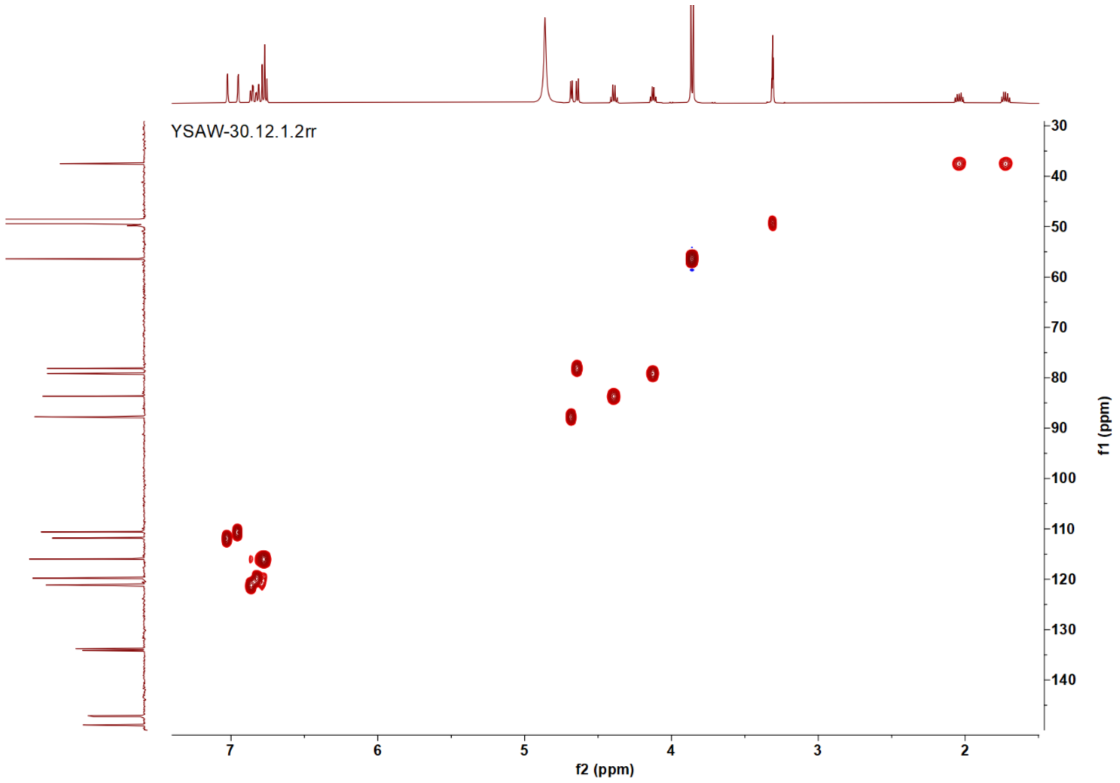

Figure S22. HSQC (600 MHz) spectrum of **3** in methanol-d_4.

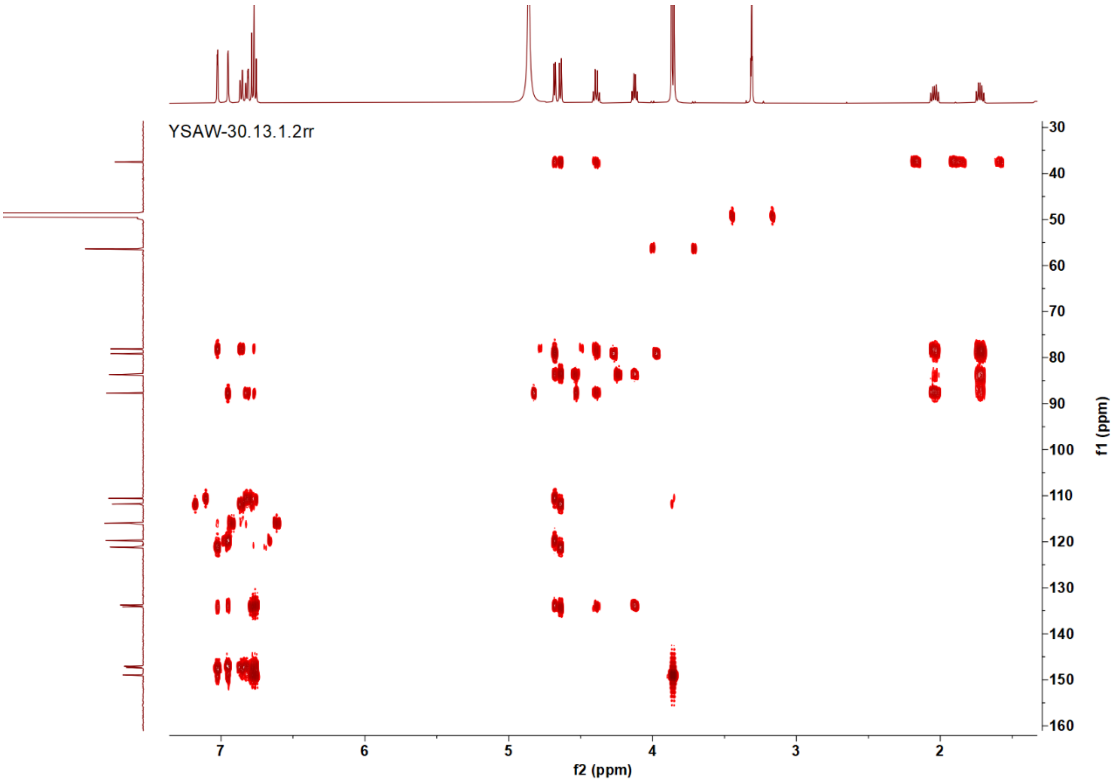

Figure S23. HMBC (600 MHz) spectrum of **3** in methanol-*d*4.

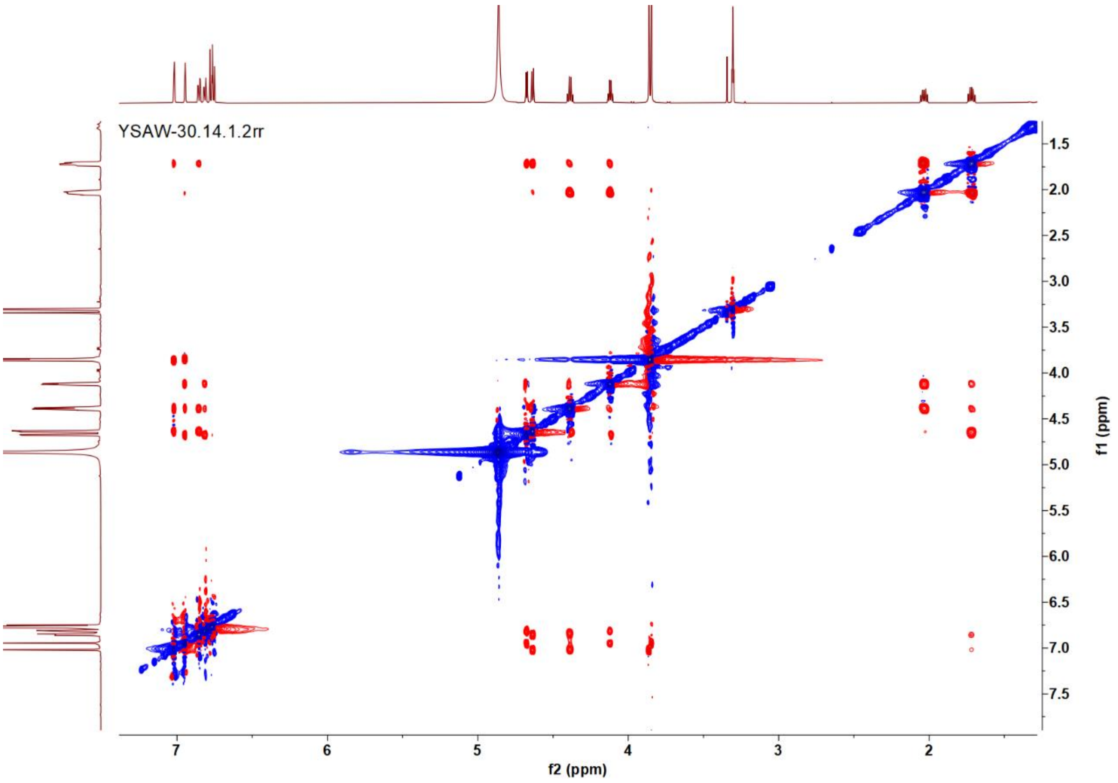

Figure S24. ROESY (600 MHz) spectrum of **3** in methanol-*d*4.

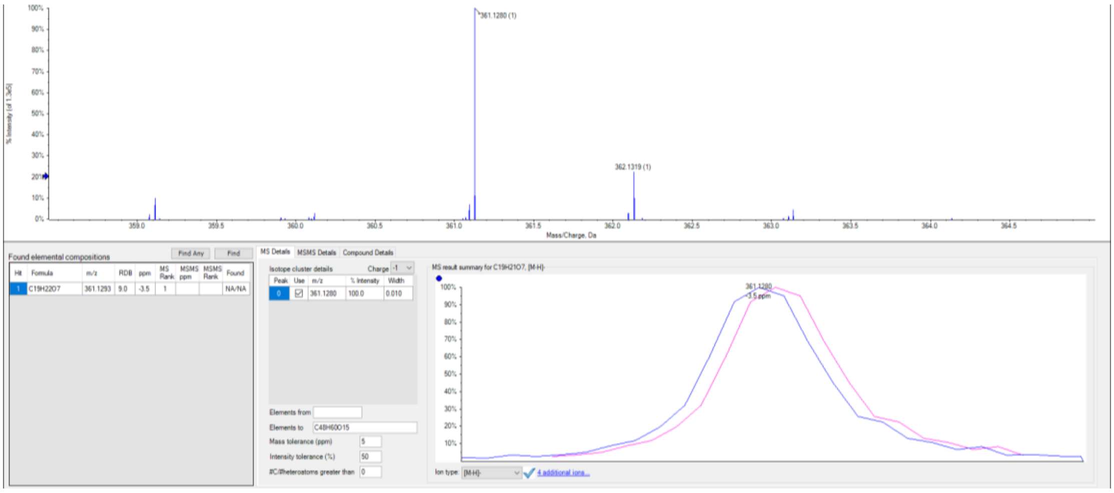

Figure S25. HR-ESI-MS spectrum of **3**.

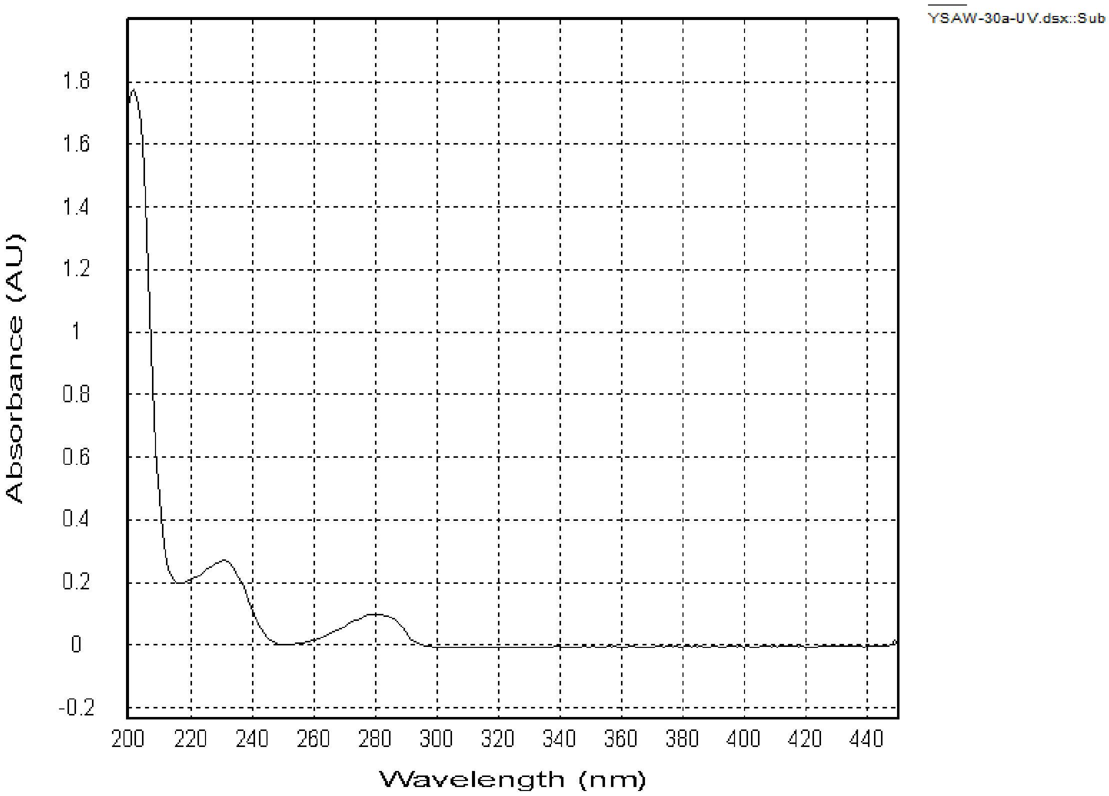

Figure S26. UV spectrum of **3.**

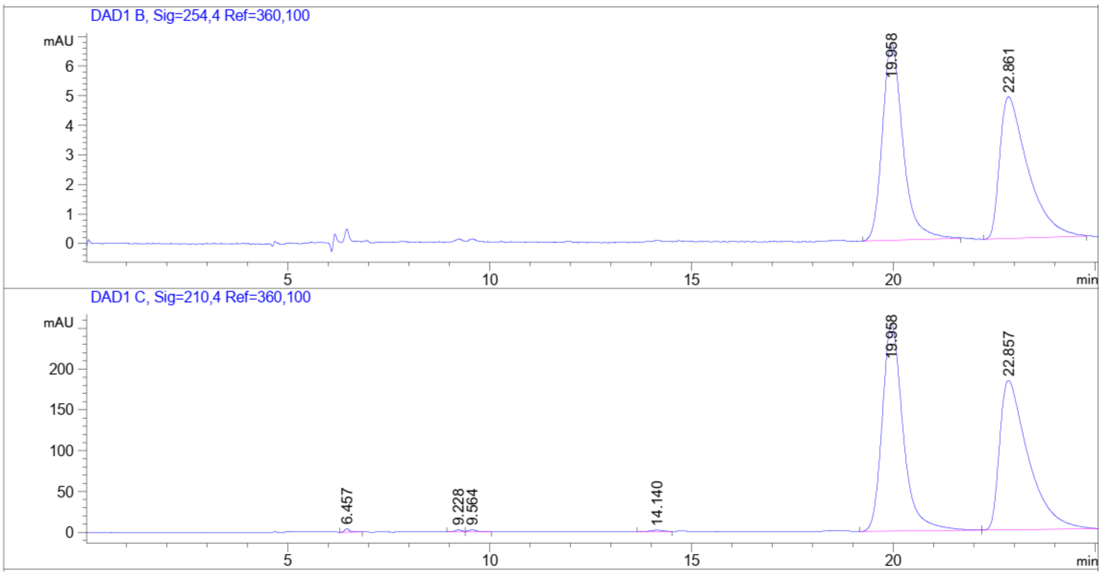

Figure S27. The HPLC chromatogram on chiral resolution of **3** by Daicel Chiralpak IC column (250 mm × 4.6 mm, i.d., 5 μm).

Analysis condition: n-hexane/EtOH 75:25, flow rate: 1.0 mL/min.

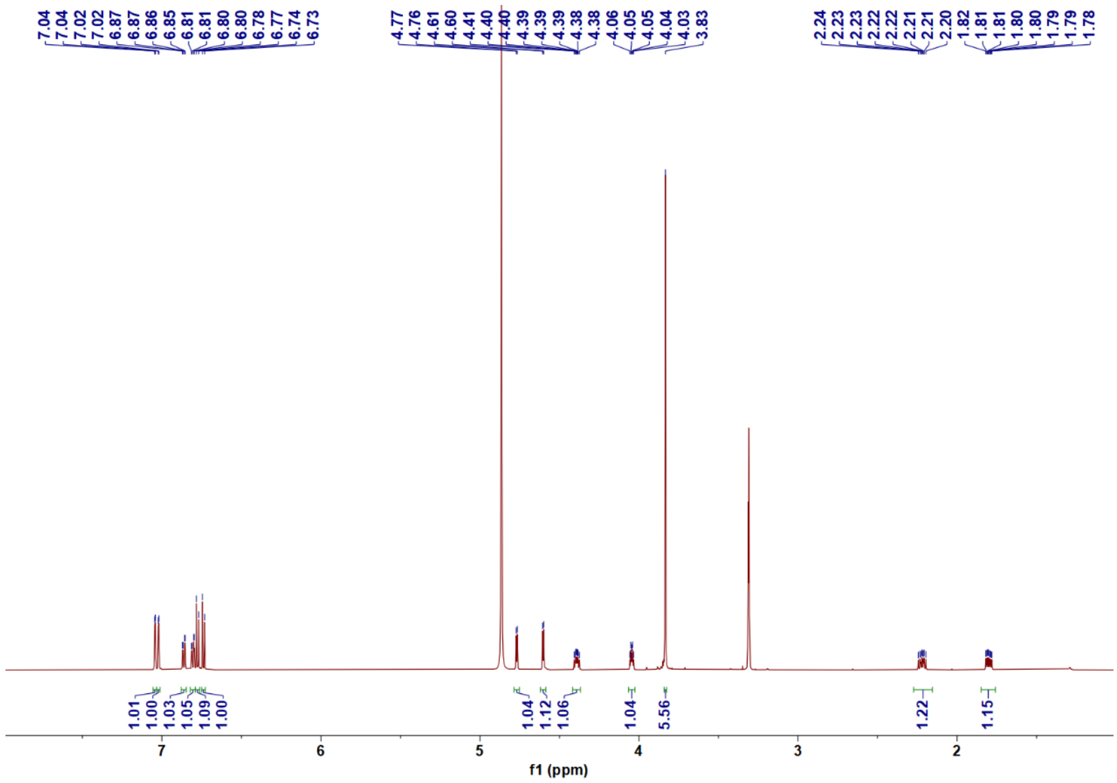

Figure S28. ^{1}H NMR (600 MHz) spectrum of **4** in methanol-*d*4.

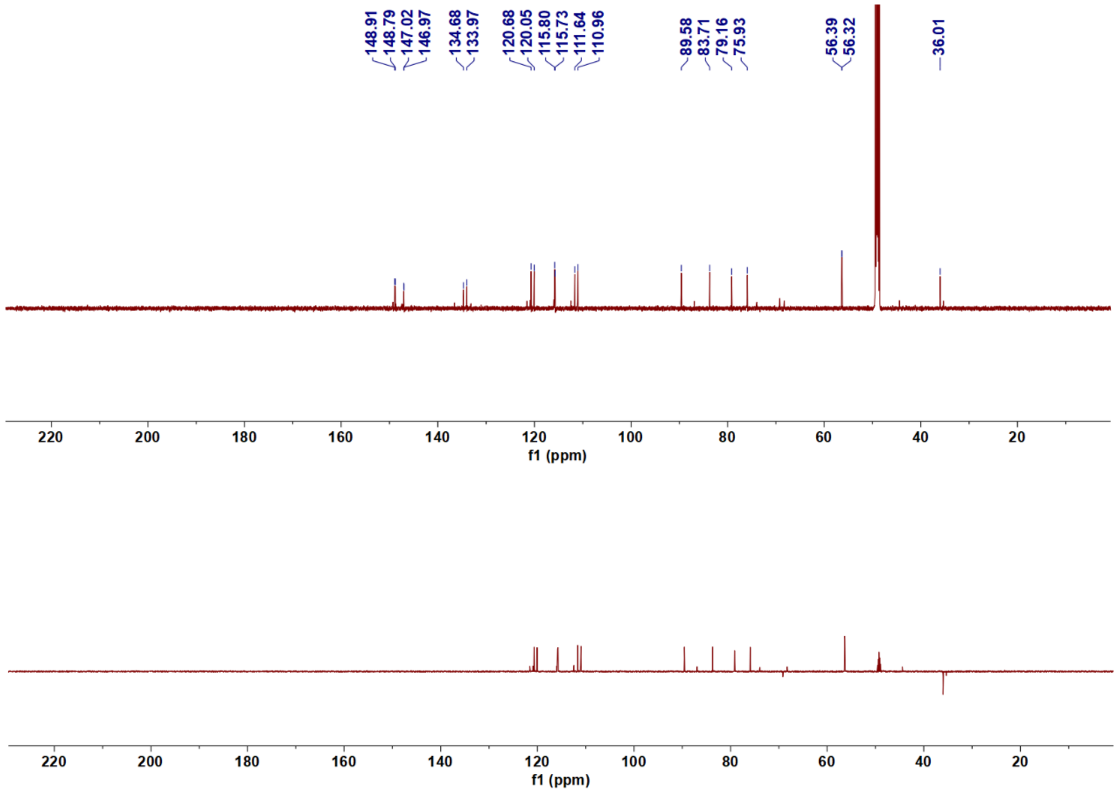

Figure S29. ^{13}C NMR and DEPT (150 MHz) spectra of **4** in methanol-*d*4.

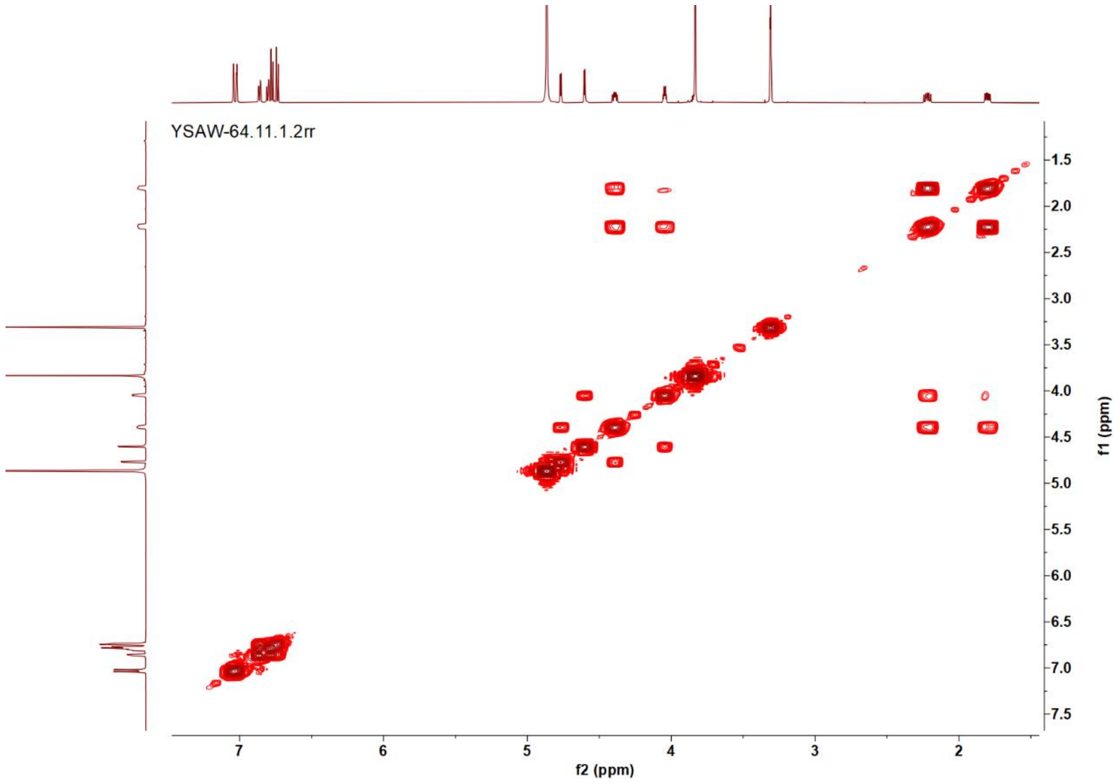

Figure S30. ^{1}H-^{1}H COSY (600 MHz) spectrum of **4** in methanol-d_4.

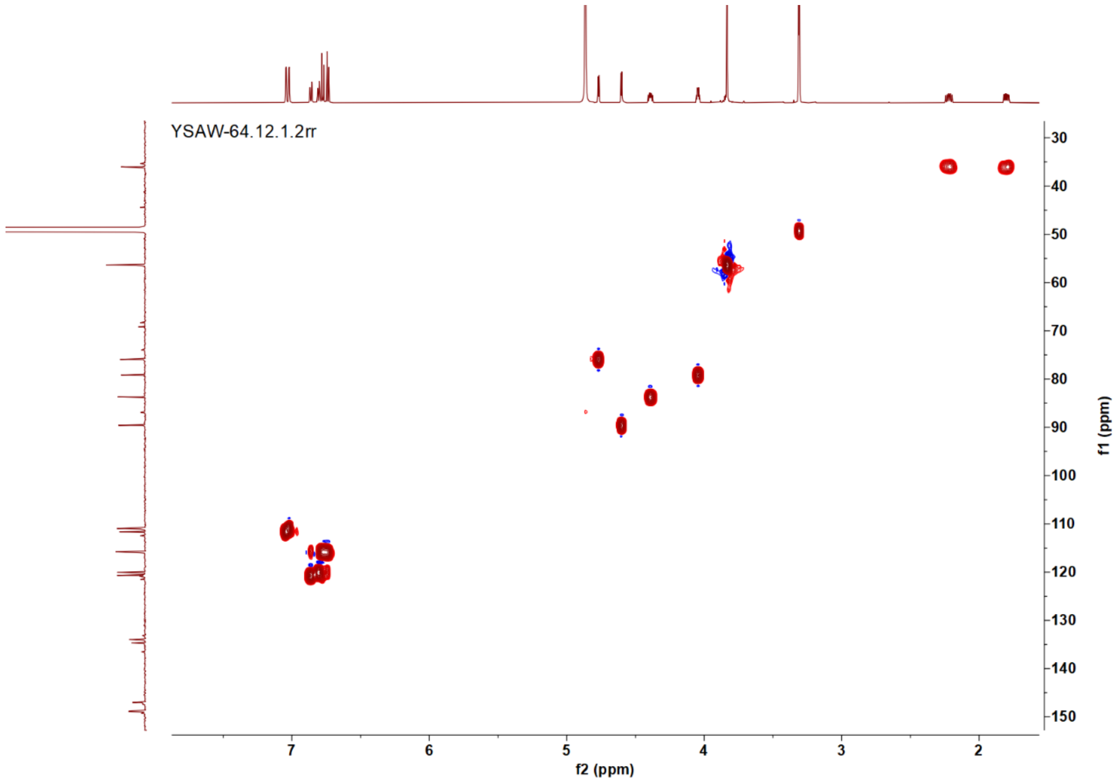

Figure S31. HSQC (600 MHz) spectrum of **4** in methanol-d_4.

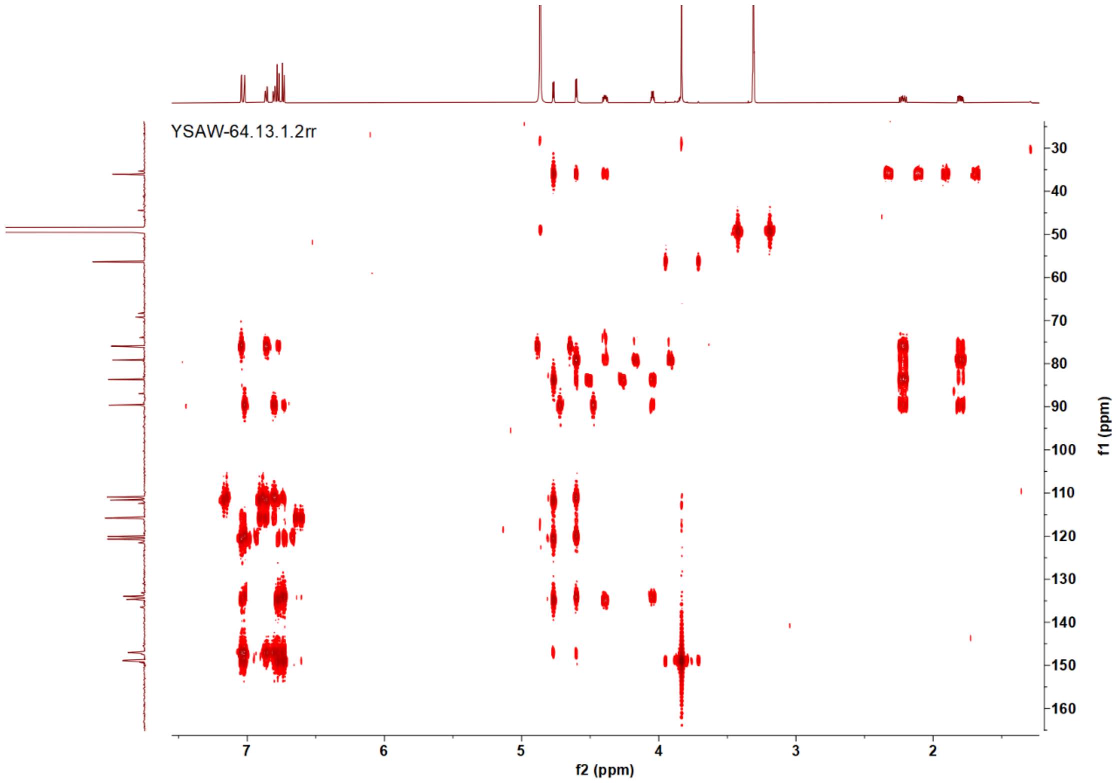

Figure S32. HMBC (600 MHz) spectrum of **4** in methanol-d_4.

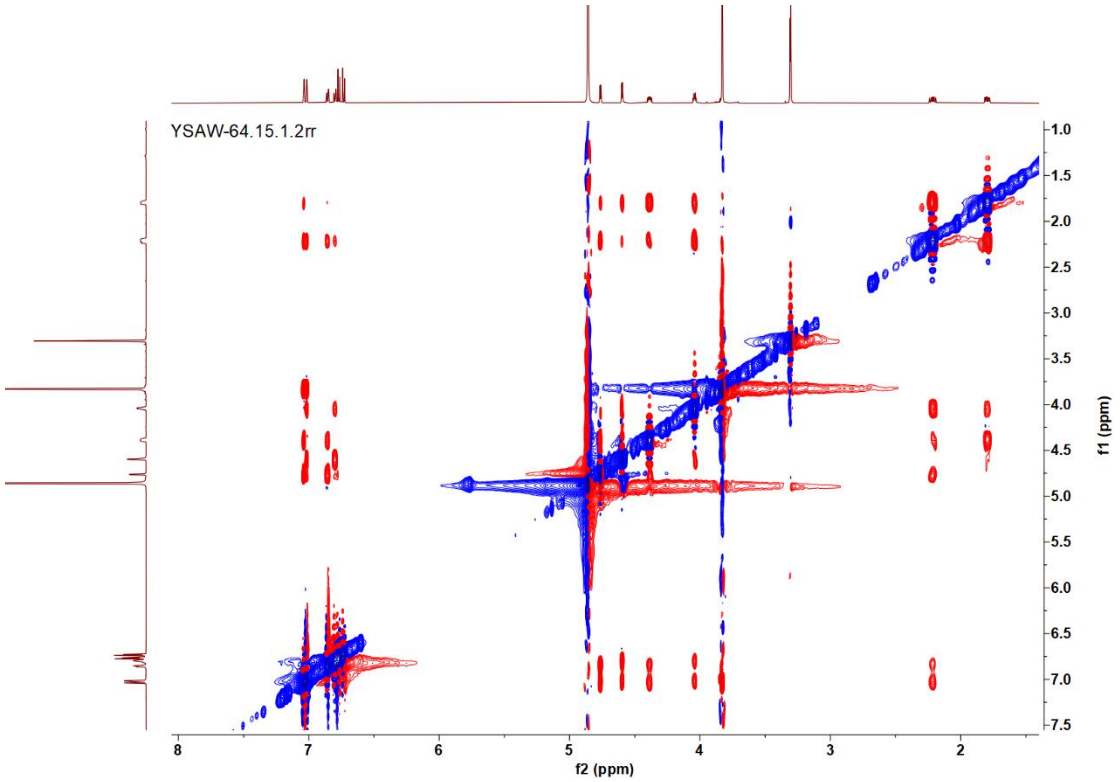

Figure S33. ROESY (600 MHz) spectrum of **4** in methanol-d_4.

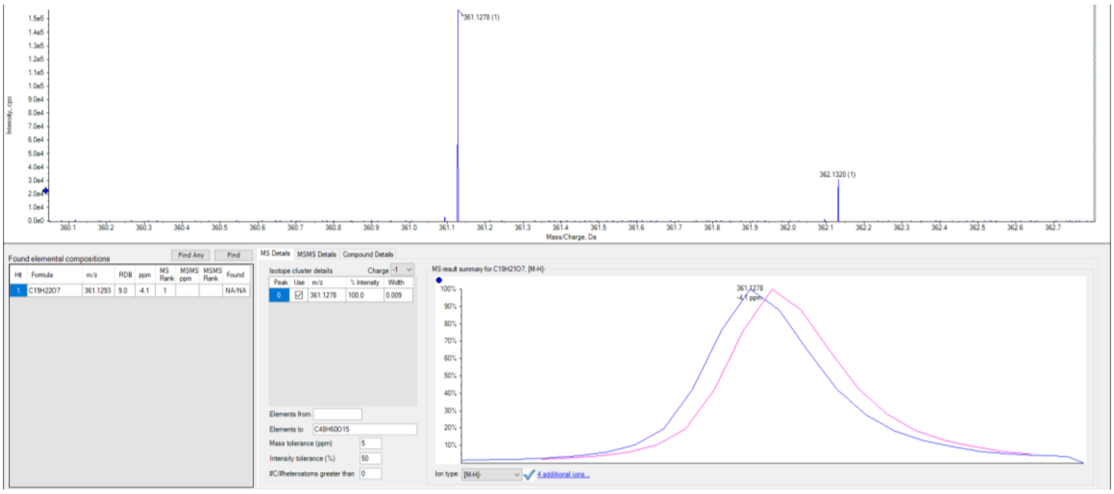

Figure S34. HR-ESI-MS spectrum of **4**.

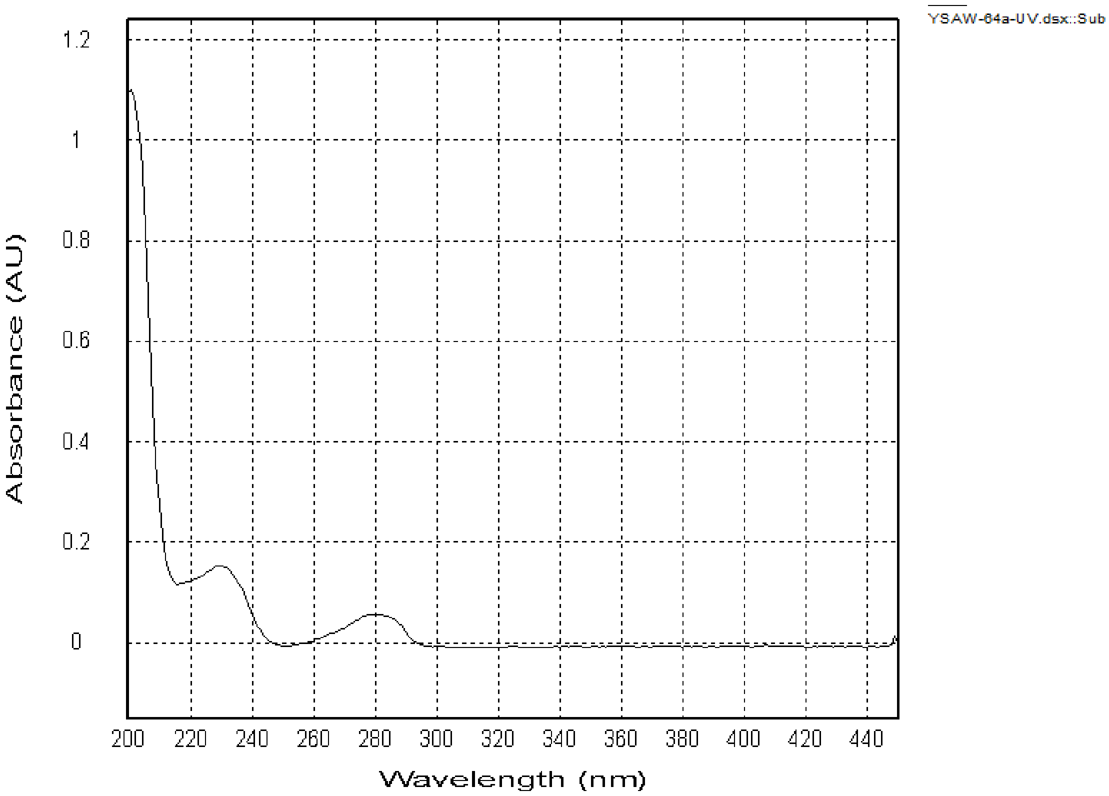

Figure S35. UV spectrum of **4.**

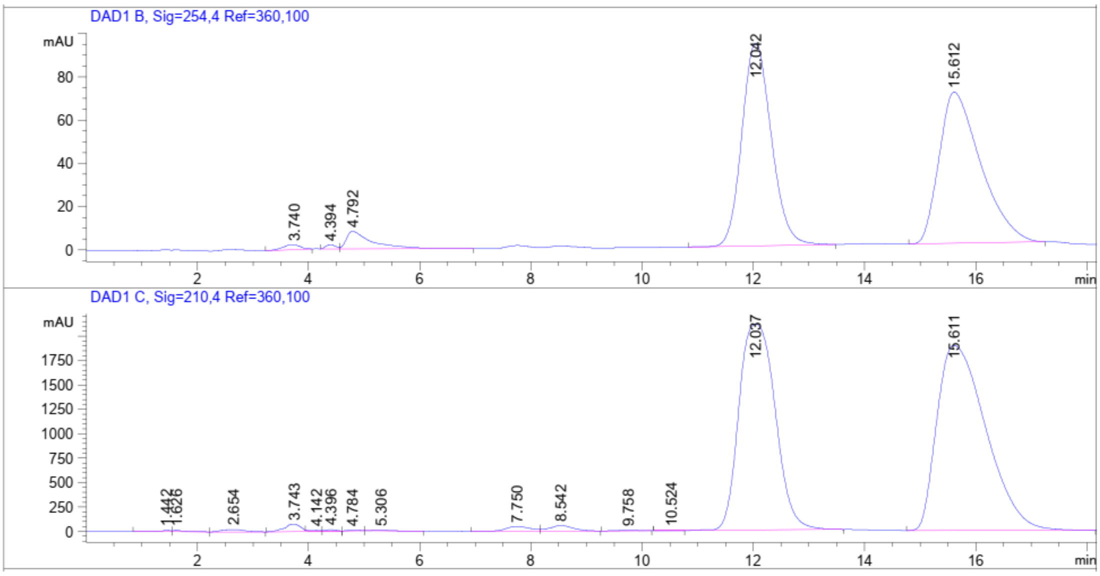

Figure S36. The HPLC chromatogram on chiral resolution of **4** by Daicel Chiralpak AD-H column (250 mm × 4.6 mm, i.d., 5 μm).

Analysis condition: n-hexane/EtOH 68:32, flow rate: 1.0 mL/min.

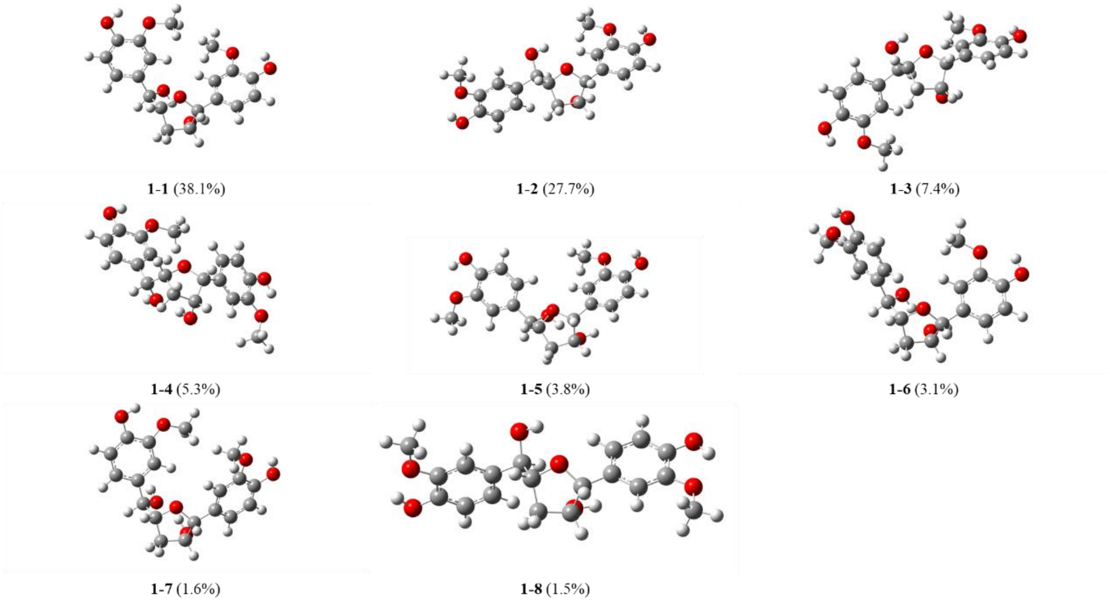

Figure S37. B3LYP/6-31G (d, p) optimized lowest energy conformers 1–8 for **1**.

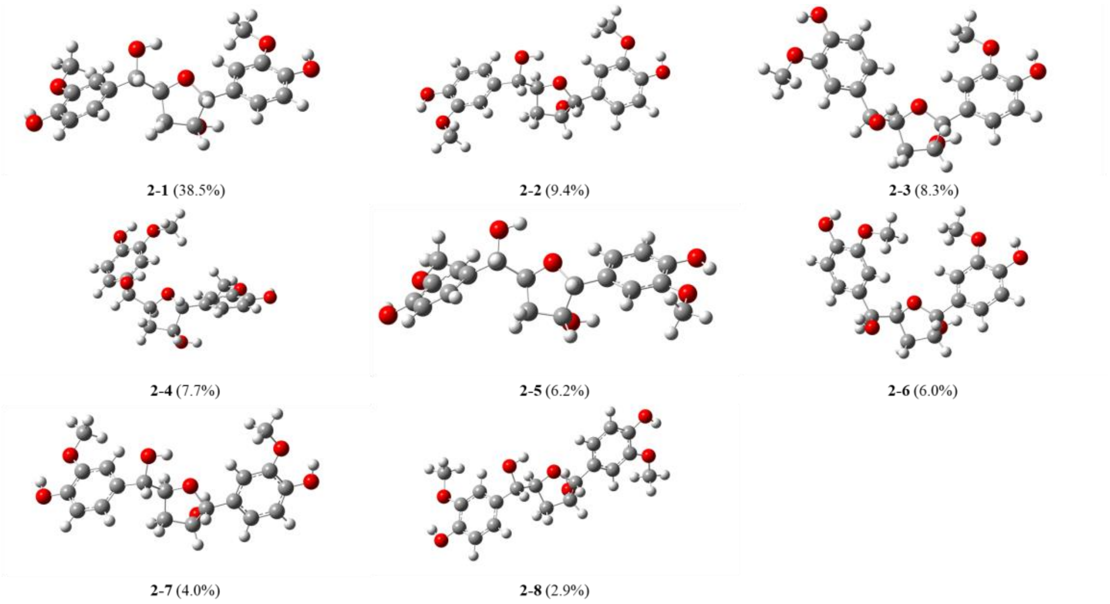

Figure S38. B3LYP/6-31G (d, p) optimized lowest energy conformers 1–8 for **2**.

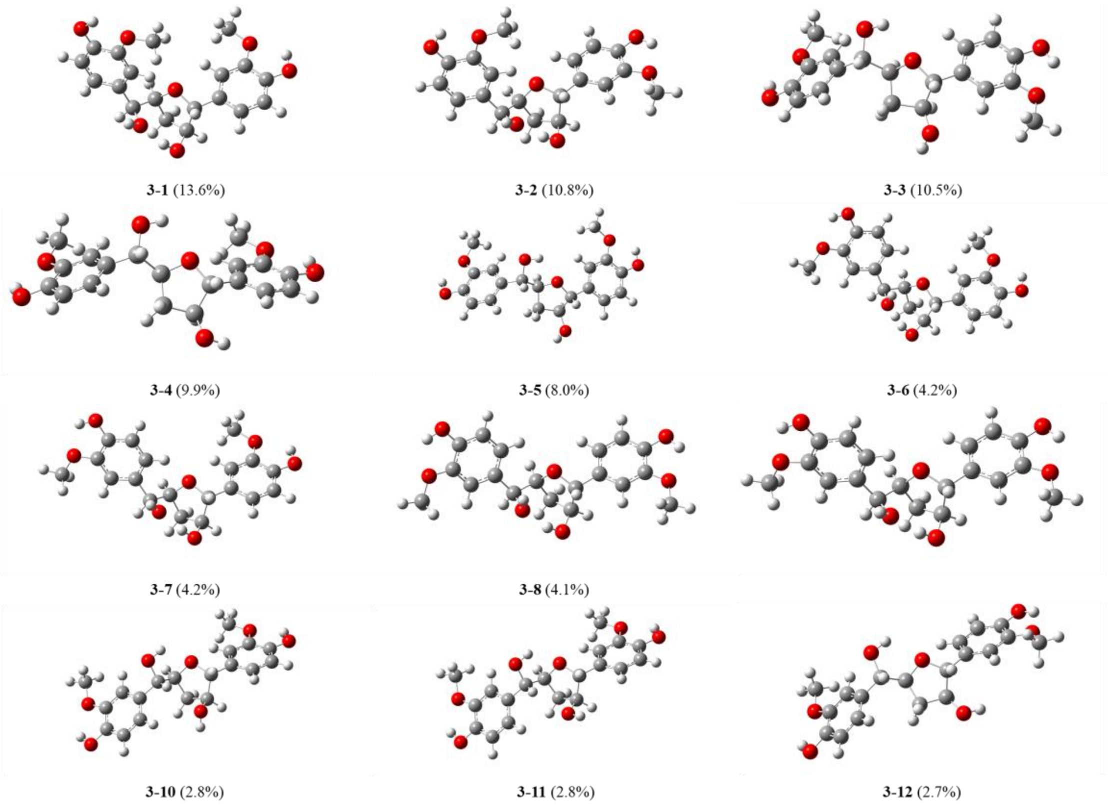

Figure S39. B3LYP/6-31G (d, p) optimized lowest energy conformers 1–12 for **3**.

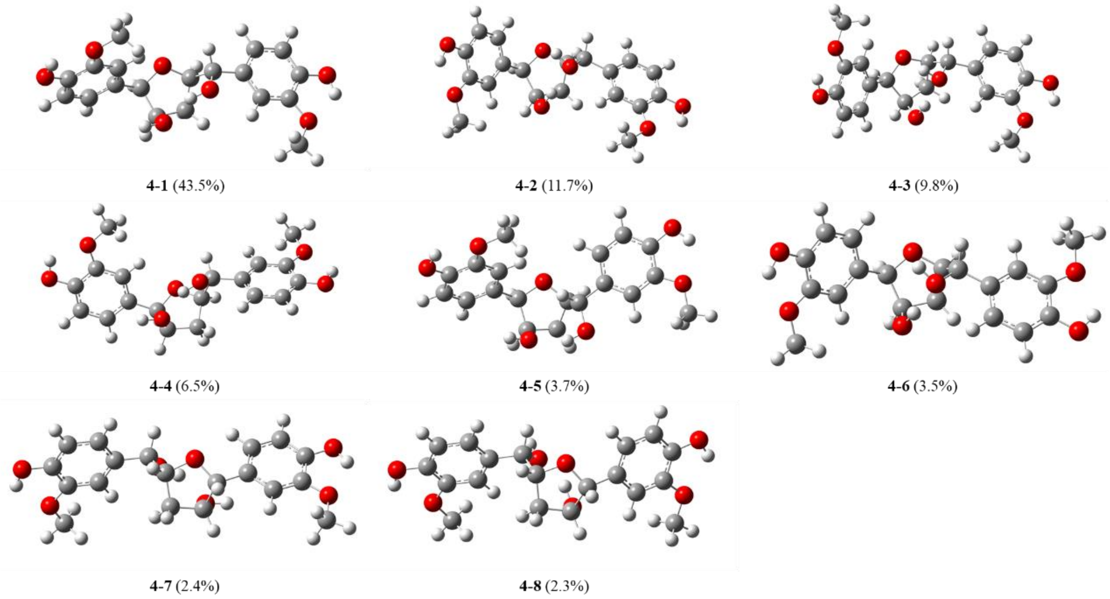

Figure S40. B3LYP/6-31G (d, p) optimized lowest energy conformers 1–8 for **4**.

Table S1. The Cartesian coordinates of the lowest energy conformers for **1**.

**1-1**	X axis(Å)	Y axis(Å)	Z axis(Å)	**1-2**	X axis(Å)	Y axis(Å)	Z axis(Å)
C	-4.9461	0.432	0.4365	C	4.0156	-0.2214	2.0655
C	-4.6823	-0.8235	-0.0934	C	5.069	0.0136	1.1927
C	-3.3764	-1.2488	-0.3555	C	4.8503	0.3698	-0.1422
C	-2.3199	-0.3842	-0.0688	C	3.5373	0.4776	-0.6024
C	-2.56	0.899	0.4613	C	2.4512	0.243	0.2639
C	-3.8849	1.2937	0.7129	C	2.7067	-0.104	1.6012
O	-5.7414	-1.649	-0.3603	O	6.3455	-0.1087	1.6719
O	-3.27	-2.511	-0.8823	O	5.9874	0.5756	-0.8813
C	-1.413	1.8505	0.7519	C	1.0242	0.3322	-0.2279
C	-0.6962	2.3299	-0.5458	C	0.5703	-0.9026	-1.0424
C	0.208	3.5472	-0.3341	C	0.4912	-2.2208	-0.2786
C	1.5778	2.9394	-0.1499	C	-0.9253	-2.2209	0.2452
C	1.5078	1.7486	-1.1166	C	-1.6768	-1.5757	-0.9305
O	0.1434	1.2893	-1.0986	O	-0.7722	-0.6231	-1.5301
C	2.4781	0.6323	-0.8042	C	-2.9962	-0.9397	-0.5557
C	2.1239	-0.4187	0.0648	C	-3.0813	0.4224	-0.2048
C	3.0381	-1.4216	0.3849	C	-4.3	0.9863	0.1739
C	4.3142	-1.3712	-0.1828	C	-5.4368	0.1729	0.2024
C	4.6883	-0.3471	-1.0429	C	-5.3783	-1.1722	-0.1356
C	3.7715	0.6577	-1.3504	C	-4.1574	-1.7292	-0.5127
O	5.2357	-2.3403	0.1073	O	-6.6471	0.6932	0.5727
O	2.8126	-2.4819	1.2245	O	-4.5102	2.2909	0.5394
C	-1.9594	-2.9364	-1.2402	C	5.8007	1.0483	-2.2106
C	1.5576	-2.4984	1.8994	C	-3.3762	3.1515	0.5087
O	-0.5366	1.2082	1.6782	O	0.9029	1.4892	-1.0773
O	1.6913	2.5671	1.2294	O	-0.9386	-1.4434	1.4446
H	-1.4557	2.5812	-1.2966	H	1.2094	-1.0114	-1.927
H	-5.9699	0.7347	0.6334	H	4.2135	-0.492	3.0981
H	-1.2902	-0.678	-0.2445	H	3.321	0.754	-1.6299
H	-4.101	2.2736	1.1321	H	1.8825	-0.2913	2.2877
H	-5.3349	-2.4653	-0.7112	H	6.9177	0.11	0.9108
H	-1.8193	2.7299	1.2665	H	0.3454	0.5268	0.6083
H	0.2039	4.1548	-1.2476	H	0.6112	-3.0493	-0.9878
H	-0.118	4.1792	0.4973	H	1.2536	-2.3263	0.4964
H	2.3931	3.6312	-0.3812	H	-1.2954	-3.2217	0.4871
H	1.6805	2.0992	-2.1427	H	-1.8523	-2.3296	-1.7093
H	1.122	-0.4246	0.4834	H	-2.1754	1.0198	-0.2296
H	5.6872	-0.3345	-1.4689	H	-6.2786	-1.7789	-0.1057
H	4.0738	1.4561	-2.0248	H	-4.1213	-2.7843	-0.7766
H	4.7716	-2.9545	0.7093	H	-6.4693	1.6367	0.7552

H	-2.0423	-3.9337	-1.6829	H	6.7903	1.2108	-2.6486
H	-1.5183	-2.2741	-1.9927	H	5.2842	0.3027	-2.8239
H	-1.318	-3.0174	-0.3569	H	5.2711	2.0068	-2.2203
H	1.5468	-3.3705	2.5603	H	-3.7065	4.149	0.814
H	1.4306	-1.6066	2.5223	H	-2.6118	2.8216	1.22
H	0.7315	-2.6086	1.1897	H	-2.9682	3.2312	-0.5043
H	0.2705	1.7617	1.7308	H	0.0731	1.3463	-1.573
H	2.5608	2.1315	1.3335	H	-1.8664	-1.3751	1.7384
**1-3**	X axis(Å)	Y axis(Å)	Z axis(Å)	**1-4**	X axis(Å)	Y axis(Å)	Z axis(Å)
C	4.8233	-0.3185	-1.5003	C	5.1184	-0.3548	0.2794
C	5.2519	0.2401	-0.3041	C	4.7499	0.9811	0.1904
C	4.3456	0.6937	0.6602	C	3.4079	1.373	0.1838
C	2.9785	0.5746	0.4062	C	2.4238	0.3892	0.2743
C	2.5192	0.0078	-0.7994	C	2.7722	-0.9743	0.3665
C	3.4565	-0.4348	-1.7471	C	4.1292	-1.3338	0.3664
O	6.5992	0.3403	-0.0829	O	5.7417	1.9206	0.107
O	4.9169	1.2188	1.7913	O	3.1958	2.7243	0.0902
C	1.0341	-0.1425	-1.0381	C	1.6983	-2.0431	0.4687
C	0.5211	-1.5923	-0.867	C	0.9352	-2.2774	-0.8627
C	0.6524	-2.1907	0.5303	C	-0.066	-3.431	-0.8002
C	-0.6427	-1.7844	1.1917	C	-1.3905	-2.7497	-0.5329
C	-1.6297	-1.9164	0.0193	C	-1.2237	-1.4231	-1.2919
O	-0.8997	-1.5811	-1.1806	O	0.1824	-1.1035	-1.2304
C	-2.8866	-1.0889	0.1677	C	-2.0446	-0.2177	-0.8783
C	-2.9967	0.1918	-0.4095	C	-3.2552	-0.3388	-0.1697
C	-4.1495	0.9574	-0.2311	C	-3.9998	0.7919	0.1772
C	-5.1934	0.4319	0.5363	C	-3.5412	2.0516	-0.2193
C	-5.1083	-0.826	1.117	C	-2.3668	2.2011	-0.9401
C	-3.9543	-1.5861	0.9339	C	-1.6248	1.0684	-1.2708
O	-6.3358	1.1585	0.7351	O	-4.2533	3.1762	0.0993
O	-4.3746	2.2122	-0.7358	O	-5.1778	0.8001	0.8808
C	4.0238	1.7109	2.7841	C	1.8382	3.1501	0.0239
C	-3.3466	2.7643	-1.552	C	-5.5982	-0.4459	1.4261
O	0.7382	0.2803	-2.3814	O	0.7952	-1.7144	1.5333
O	-0.4783	-0.4476	1.6732	O	-1.5712	-2.6186	0.876
H	1.0027	-2.2411	-1.6084	H	1.6658	-2.4664	-1.6585
H	5.5476	-0.6555	-2.2354	H	6.1694	-0.6284	0.2805
H	2.2412	0.9067	1.1317	H	1.3686	0.6501	0.2722
H	3.1203	-0.8613	-2.6902	H	4.4292	-2.3771	0.4359
H	6.676	0.7657	0.7931	H	5.2712	2.7753	0.0498
H	0.4747	0.5495	-0.4007	H	2.1734	-2.9872	0.7621
H	0.6843	-3.2841	0.4462	H	-0.1053	-3.9136	-1.7848
H	1.5479	-1.8664	1.0652	H	0.2045	-4.1903	-0.0602

H	-0.9095	-2.4182	2.0427	H	-2.2404	-3.3272	-0.9094
H	-1.9108	-2.9706	-0.1046	H	-1.4379	-1.61	-2.3532
H	-2.1621	0.5707	-0.9906	H	-3.6016	-1.3281	0.1107
H	-5.9369	-1.2092	1.7054	H	-2.0351	3.1886	-1.2447
H	-3.8972	-2.5717	1.3916	H	-0.707	1.1891	-1.8441
H	-6.195	1.9885	0.2386	H	-5.03	2.8458	0.5916
H	4.626	2.1039	3.6089	H	1.8353	4.2405	-0.0674
H	3.4149	2.5337	2.3951	H	1.3394	2.7409	-0.8608
H	3.3968	0.9069	3.1836	H	1.2982	2.8915	0.9407
H	-3.682	3.7497	-1.8893	H	-6.5032	-0.2638	2.0137
H	-2.4229	2.9044	-0.9807	H	-5.8547	-1.1548	0.6321
H	-3.1743	2.1485	-2.441	H	-4.8415	-0.8606	2.1005
H	-0.1398	-0.1001	-2.5769	H	1.3046	-1.2012	2.1879
H	-1.3468	-0.1482	2.0022	H	-0.8151	-2.0929	1.2232
**1-5**	X axis(Å)	Y axis(Å)	Z axis(Å)	**1-6**	X axis(Å)	Y axis(Å)	Z axis(Å)
C	-2.8559	-1.7667	-1.0303	C	-3.2263	-0.6324	-1.9524
C	-4.1937	-1.5656	-0.7238	C	-4.2839	-0.9824	-1.126
C	-4.6173	-0.4534	0.0109	C	-4.3618	-0.5262	0.1921
C	-3.6658	0.4779	0.4331	C	-3.3536	0.3118	0.6713
C	-2.301	0.3013	0.1266	C	-2.2716	0.6987	-0.1519
C	-1.9113	-0.836	-0.6006	C	-2.2191	0.2014	-1.4635
O	-5.1035	-2.4929	-1.1556	O	-5.2573	-1.8018	-1.6305
O	-5.9683	-0.3942	0.2422	O	-5.4552	-0.9645	0.8944
C	-1.2789	1.3237	0.5927	C	-1.2071	1.6092	0.4422
C	-0.5274	2.0189	-0.5829	C	-0.2755	2.3491	-0.5669
C	0.2078	3.297	-0.1695	C	0.5601	3.4678	0.0614
C	1.6194	2.8236	0.0812	C	1.8823	2.7998	0.3558
C	1.7695	1.746	-1.002	C	2.0039	1.8302	-0.8288
O	0.4695	1.149	-1.1642	O	0.6636	1.4415	-1.181
C	2.8333	0.7135	-0.7052	C	2.8826	0.6292	-0.5619
C	2.5131	-0.4997	-0.0647	C	2.3287	-0.6038	-0.1633
C	3.5058	-1.4291	0.2438	C	3.1474	-1.696	0.121
C	4.8292	-1.1343	-0.0961	C	4.532	-1.545	0.0023
C	5.1713	0.0543	-0.7274	C	5.1027	-0.3409	-0.3891
C	4.1734	0.9799	-1.0301	C	4.2779	0.7477	-0.6697
O	5.8286	-2.0229	0.1943	O	5.3646	-2.596	0.2764
O	3.3179	-2.6348	0.8694	O	2.7274	-2.9383	0.5224
C	-6.4295	0.6798	1.053	C	-5.5203	-0.5882	2.2652
C	1.9786	-2.9541	1.2375	C	1.3195	-3.116	0.6553
O	-0.4112	0.6724	1.5193	O	-0.4965	0.8196	1.3967
O	1.6621	2.3145	1.4203	O	1.7536	2.1556	1.6291
H	-1.2537	2.268	-1.3665	H	-0.8951	2.7822	-1.3618
H	-2.5499	-2.6415	-1.5954	H	-3.1808	-1.0123	-2.9686

H	-3.9587	1.3498	1.0084	H	-3.3678	0.6776	1.6938
H	-0.8622	-1.0035	-0.8335	H	-1.3912	0.4414	-2.1255
H	-5.9642	-2.1672	-0.8285	H	-5.8834	-1.9265	-0.8911
H	-1.8092	2.1002	1.1577	H	-1.7419	2.3866	1.0041
H	0.2075	3.9955	-1.0156	H	0.7155	4.2555	-0.6864
H	-0.2561	3.797	0.6859	H	0.0791	3.9167	0.9355
H	2.3645	3.6204	-0.001	H	2.7178	3.5032	0.4188
H	1.9915	2.2218	-1.9663	H	2.3886	2.3671	-1.7057
H	1.4741	-0.6905	0.1884	H	1.2475	-0.681	-0.0785
H	6.2081	0.2541	-0.9817	H	6.1819	-0.2555	-0.4756
H	4.4504	1.9076	-1.5263	H	4.7331	1.6865	-0.9778
H	5.3738	-2.7801	0.6131	H	4.7656	-3.3316	0.5122
H	-7.5115	0.5677	1.1714	H	-6.4215	-1.0389	2.6922
H	-5.98	0.6441	2.0509	H	-4.6598	-0.9727	2.8229
H	-6.246	1.6448	0.5693	H	-5.6095	0.4979	2.3714
H	1.9922	-3.9347	1.7231	H	1.1438	-4.1465	0.9785
H	1.5844	-2.2309	1.9592	H	0.9085	-2.452	1.4231
H	1.3339	-3.0285	0.3555	H	0.8116	-2.9774	-0.3049
H	0.3347	1.2853	1.6862	H	0.2954	1.3292	1.6657
H	2.5585	1.9465	1.5549	H	2.5777	1.6495	1.7777
**1-7**	X axis(Å)	Y axis(Å)	Z axis(Å)	**1-8**	X axis(Å)	Y axis(Å)	Z axis(Å)
C	4.7769	-0.2337	0.326	C	4.2946	-1.4515	1.492
C	4.1739	-1.453	0.044	C	5.3489	-0.8791	0.7938
C	2.8342	-1.5359	-0.3467	C	5.153	0.1965	-0.0789
C	2.0935	-0.3589	-0.4454	C	3.8602	0.6908	-0.2564
C	2.6847	0.8922	-0.1708	C	2.7733	0.1255	0.4393
C	4.0322	0.9405	0.2181	C	3.0073	-0.9468	1.3167
O	4.9298	-2.5885	0.1559	O	6.604	-1.3926	0.9817
O	2.3745	-2.8038	-0.5945	O	6.2891	0.6606	-0.6919
C	1.8734	2.1702	-0.2846	C	1.3638	0.6352	0.2366
C	0.9395	2.4109	0.9309	C	0.7126	0.1489	-1.0798
C	0.1307	3.7062	0.837	C	0.4801	-1.3552	-1.1991
C	-1.2255	3.2536	0.3365	C	-0.9122	-1.5246	-0.6394
C	-1.3551	1.8736	0.999	C	-1.5969	-0.256	-1.1719
O	-0.0167	1.3381	1.0571	O	-0.5952	0.782	-1.1745
C	-2.2905	0.8328	0.4198	C	-2.8398	0.1521	-0.4139
C	-2.1568	-0.5117	0.8349	C	-4.0828	-0.4157	-0.7604
C	-3.0146	-1.5037	0.3585	C	-5.2397	-0.0792	-0.0546
C	-4.0134	-1.1463	-0.5499	C	-5.1418	0.8242	1.0085
C	-4.1807	0.1681	-0.9613	C	-3.9289	1.3931	1.3699
C	-3.3277	1.1571	-0.4676	C	-2.7785	1.0568	0.6587
O	-4.8647	-2.0952	-1.0475	O	-6.2556	1.1694	1.7246
O	-2.9733	-2.834	0.6894	O	-6.5023	-0.558	-0.2918

C	1.0468	-2.9071	-1.1003	C	6.1444	1.8338	-1.4847
C	-2.164	-3.1789	1.8089	C	-6.6589	-1.4293	-1.4055
O	1.133	2.1556	-1.5126	O	1.3995	2.0739	0.204
O	-1.2135	3.2385	-1.0889	O	-0.7966	-1.5769	0.7838
H	1.5469	2.4186	1.8441	H	1.299	0.5121	-1.9325
H	5.8199	-0.2016	0.627	H	4.4752	-2.2817	2.1678
H	1.0429	-0.3783	-0.7246	H	3.6619	1.5255	-0.922
H	4.5138	1.8901	0.441	H	2.1818	-1.3987	1.8644
H	4.3227	-3.3167	-0.0807	H	7.1832	-0.8414	0.4203
H	2.563	3.0196	-0.3643	H	0.7407	0.3871	1.1018
H	0.0191	4.1231	1.8457	H	0.4763	-1.6304	-2.2612
H	0.6101	4.4628	0.2085	H	1.2388	-1.9596	-0.6973
H	-2.0313	3.9277	0.6422	H	-1.4033	-2.4435	-0.9732
H	-1.6589	2.0295	2.0437	H	-1.859	-0.4004	-2.2287
H	-1.3582	-0.7531	1.5312	H	-4.1203	-1.1172	-1.588
H	-4.9711	0.4237	-1.6607	H	-3.8804	2.0933	2.1987
H	-3.4825	2.1816	-0.7995	H	-1.8301	1.5033	0.951
H	-4.5781	-2.9308	-0.631	H	-6.9879	0.6751	1.308
H	0.8409	-3.9662	-1.2825	H	7.1365	2.1102	-1.8547
H	0.318	-2.5477	-0.3679	H	5.5066	1.6439	-2.3542
H	0.9445	-2.3781	-2.0537	H	5.7659	2.6714	-0.8894
H	-2.3272	-4.2395	2.0234	H	-7.7184	-1.6935	-1.4764
H	-1.1019	-3.046	1.5821	H	-6.376	-0.9323	-2.3394
H	-2.4524	-2.6106	2.6997	H	-6.0933	-2.3558	-1.2623
H	1.6983	1.7167	-2.1753	H	0.5209	2.3355	-0.1338
H	-0.4755	2.6553	-1.3754	H	-1.6968	-1.5391	1.1571

Table S2. The Cartesian coordinates of the lowest energy conformers for **2**

**2-1**	X axis(Å)	Y axis(Å)	Z axis(Å)	**2-2**	X axis(Å)	Y axis(Å)	Z axis(Å)
C	-5.0676	-1.5684	0.1458	C	4.3453	1.9223	0.8096
C	-5.5347	-0.3556	-0.3416	C	5.3432	0.9626	0.7186
C	-4.7665	0.8106	-0.2656	C	5.1588	-0.2207	-0.0046
C	-3.4965	0.7414	0.3082	C	3.934	-0.4388	-0.6389
C	-2.9991	-0.4785	0.8087	C	2.9039	0.5187	-0.5561
C	-3.7999	-1.6295	0.7222	C	3.1271	1.7007	0.1703
O	-6.7823	-0.3226	-0.904	O	6.5308	1.2019	1.3561
O	-5.3622	1.9319	-0.7842	O	6.2357	-1.0702	-0.0074
C	-1.612	-0.5665	1.4036	C	1.5716	0.2594	-1.2221
C	-0.4962	-0.5662	0.3438	C	0.4301	-0.0046	-0.2241
C	-0.5059	-1.765	-0.5972	C	0.6055	-1.2563	0.6274
C	0.9417	-1.8152	-1.0293	C	-0.8282	-1.5751	0.9852
C	1.653	-1.4937	0.297	C	-1.5373	-1.2675	-0.3459

O	0.7886	-0.5909	1.0234	O	-0.8039	-0.1891	-0.9694
C	3.053	-0.9442	0.144	C	-3.0107	-0.9545	-0.2185
C	3.3049	0.4416	0.171	C	-3.4744	0.3736	-0.1422
C	4.5992	0.9333	-0.0028	C	-4.8349	0.6452	0.0071
C	5.6417	0.0247	-0.2106	C	-5.7299	-0.4264	0.0851
C	5.4181	-1.3447	-0.245	C	-5.297	-1.7434	0.0164
C	4.1233	-1.8295	-0.069	C	-3.9368	-2.0078	-0.1339
O	6.9219	0.4741	-0.3889	O	-7.0703	-0.1948	0.2357
O	4.9728	2.2526	0.0014	O	-5.409	1.8873	0.0948
C	-4.636	3.1496	-0.6596	C	6.1202	-2.2443	-0.8023
C	3.9423	3.2042	0.2437	C	-4.5299	3.0024	-0.0057
O	-1.4074	0.5515	2.2796	O	1.2284	1.3963	-2.0249
O	1.1199	-0.8168	-2.0379	O	-1.2025	-0.705	2.0565
H	-0.5148	0.373	-0.2244	H	0.2662	0.877	0.4091
H	-5.6887	-2.4564	0.0782	H	4.5168	2.8368	1.369
H	-2.8667	1.6221	0.3916	H	3.7539	-1.346	-1.2062
H	-3.4425	-2.5828	1.1049	H	2.3498	2.4601	0.2304
H	-6.9116	0.61	-1.1653	H	7.083	0.4215	1.1567
H	-1.5252	-1.4565	2.0386	H	1.6503	-0.5809	-1.9225
H	-1.2092	-1.6278	-1.4239	H	1.2472	-1.0723	1.4943
H	-0.7633	-2.6824	-0.0549	H	1.0307	-2.0737	0.033
H	1.2368	-2.781	-1.4495	H	-0.974	-2.6069	1.3178
H	1.6965	-2.4	0.9156	H	-1.4159	-2.12	-1.0273
H	2.4664	1.1144	0.3246	H	-2.7459	1.1773	-0.1969
H	6.2481	-2.0264	-0.4062	H	-6.0161	-2.5549	0.0783
H	3.9573	-2.9045	-0.096	H	-3.6064	-3.0431	-0.188
H	6.8578	1.4468	-0.3206	H	-7.1557	0.7786	0.2528
H	-5.2493	3.9485	-1.0874	H	7.0661	-2.7899	-0.7313
H	-4.454	3.3964	0.3916	H	5.3289	-2.8994	-0.4232
H	-3.6999	3.1094	-1.2262	H	5.9578	-1.9949	-1.8561
H	4.3982	4.1989	0.2342	H	-5.1348	3.9113	0.0672
H	3.1867	3.1768	-0.5484	H	-3.8116	3.0135	0.8207
H	3.4899	3.0547	1.2297	H	-4.0208	3.0182	-0.9751
H	-0.4513	0.5397	2.4802	H	0.2876	1.2693	-2.2543
H	2.0746	-0.7521	-2.2245	H	-2.1616	-0.8049	2.1991
**2-3**	X axis(Å)	Y axis(Å)	Z axis(Å)	**2-4**	X axis(Å)	Y axis(Å)	Z axis(Å)
C	2.9017	1.971	0.4392	C	-4.8596	-0.0579	1.0189
C	4.2534	1.8561	0.1492	C	-4.4954	-1.2568	0.4227
C	4.8362	0.6192	-0.1373	C	-3.3519	-1.3597	-0.3749
C	4.0304	-0.5201	-0.1195	C	-2.5634	-0.2241	-0.5685
C	2.6519	-0.4353	0.1803	C	-2.9037	1.0041	0.0349
C	2.1018	0.8272	0.4513	C	-4.0649	1.0714	0.8239
O	5.0131	2.9944	0.144	O	-5.2896	-2.3515	0.6327

O	6.1788	0.6601	-0.4129	O	-3.1171	-2.6069	-0.8954
C	1.8404	-1.7201	0.1803	C	-2.0445	2.2433	-0.1439
C	0.3809	-1.6449	0.6733	C	-0.6014	2.0729	0.3711
C	-0.2619	-3.0223	0.8381	C	0.1524	3.3944	0.4958
C	-1.7281	-2.6857	0.71	C	1.5867	2.9176	0.4774
C	-1.686	-1.6571	-0.432	C	1.5156	1.8238	-0.6027
O	-0.4426	-0.9361	-0.289	O	0.178	1.2774	-0.5591
C	-2.883	-0.7346	-0.4918	C	2.5596	0.7373	-0.484
C	-2.817	0.5901	-0.0177	C	2.2867	-0.4672	0.1952
C	-3.9397	1.4179	-0.0582	C	3.2657	-1.4531	0.3191
C	-5.1352	0.9056	-0.5717	C	4.5264	-1.2213	-0.2391
C	-5.2261	-0.3966	-1.0433	C	4.8212	-0.0407	-0.9078
C	-4.0999	-1.2168	-1.0036	C	3.8381	0.9401	-1.0289
O	-6.256	1.6893	-0.6202	O	5.5108	-2.166	-0.1335
O	-4.0034	2.719	0.3701	O	3.123	-2.6613	0.9514
C	6.7933	-0.5754	-0.7611	C	-1.9669	-2.7445	-1.7227
C	-2.7896	3.2919	0.8446	C	1.8527	-2.9264	1.5375
O	1.8496	-2.2297	-1.1595	O	-2.0313	2.603	-1.5278
O	-2.15	-2.1411	1.9644	O	1.886	2.4249	1.7867
H	0.3241	-1.0997	1.6239	H	-0.6017	1.5493	1.3361
H	2.4731	2.946	0.6502	H	-5.7563	-0.0039	1.6287
H	4.4407	-1.501	-0.3441	H	-1.6733	-0.2698	-1.1865
H	1.044	0.9487	0.669	H	-4.3623	2.0073	1.2924
H	5.9131	2.6915	-0.0861	H	-4.8692	-3.0668	0.1172
H	2.3738	-2.4412	0.8127	H	-2.5121	3.0758	0.3951
H	0.008	-3.4771	1.7961	H	-0.1255	3.9309	1.4079
H	0.0349	-3.6992	0.0287	H	-0.0368	4.0386	-0.3708
H	-2.3565	-3.5522	0.4848	H	2.3056	3.7066	0.2384
H	-1.6018	-2.1807	-1.3937	H	1.5958	2.2867	-1.595
H	-1.8735	0.9485	0.3824	H	1.2974	-0.6048	0.622
H	-6.1672	-0.7658	-1.4404	H	5.8097	0.1104	-1.3318
H	-4.1789	-2.2352	-1.379	H	4.077	1.8613	-1.5565
H	-5.9742	2.5516	-0.2567	H	5.1001	-2.9025	0.3602
H	7.8483	-0.3754	-0.9714	H	-1.9212	-3.7847	-2.0594
H	6.7489	-1.2857	0.0711	H	-2.0418	-2.1099	-2.6119
H	6.3473	-0.9974	-1.6679	H	-1.0499	-2.5361	-1.1615
H	-2.9961	4.3312	1.1177	H	1.8986	-3.9181	1.9978
H	-2.4357	2.7757	1.743	H	1.6239	-2.203	2.3271
H	-2.023	3.2991	0.0626	H	1.0644	-2.9482	0.7778
H	1.3309	-1.5798	-1.6725	H	-1.3873	1.9996	-1.9429
H	-3.0436	-1.7716	1.8399	H	2.7602	1.9951	1.748
**2-5**	X axis(Å)	Y axis(Å)	Z axis(Å)	**2-6**	X axis(Å)	Y axis(Å)	Z axis(Å)
C	-5.0314	-1.8454	0.1213	C	4.7801	-0.1887	-1.1754

C	-5.6589	-0.7098	-0.3722	C	4.461	-1.3315	-0.4543
C	-5.0639	0.553	-0.287	C	3.389	-1.3565	0.4426
C	-3.8043	0.6628	0.3031	C	2.6347	-0.1962	0.6169
C	-3.1459	-0.4755	0.8097	C	2.9327	0.9748	-0.1092
C	-3.775	-1.728	0.7135	C	4.0146	0.9643	-1.0044
O	-6.8915	-0.8522	-0.9504	O	5.2264	-2.4502	-0.6438
O	-5.8053	1.5799	-0.8133	O	3.1882	-2.5551	1.0777
C	-1.7677	-0.3668	1.4216	C	2.104	2.2355	0.0705
C	-0.6522	-0.1915	0.3761	C	0.6515	2.095	-0.436
C	-0.4798	-1.3661	-0.5801	C	-0.1251	3.4109	-0.3834
C	0.9641	-1.1998	-0.996	C	-1.552	2.9201	-0.3322
C	1.6073	-0.8006	0.3441	C	-1.4068	1.7179	0.6143
O	0.6156	-0.0427	1.0715	O	-0.1098	1.1478	0.3529
C	2.9188	-0.0586	0.2214	C	-2.5077	0.6869	0.5006
C	4.1147	-0.7834	0.0432	C	-2.3283	-0.4986	-0.2396
C	5.3336	-0.1182	-0.1031	C	-3.3601	-1.4305	-0.3539
C	5.3457	1.2802	-0.0779	C	-4.5797	-1.1631	0.2755
C	4.1808	2.0146	0.0931	C	-4.7824	-8E-4	1.0072
C	2.9679	1.3447	0.2424	C	-3.7464	0.925	1.1193
O	6.5231	1.962	-0.2245	O	-5.6138	-2.0546	0.1801
O	6.5606	-0.7036	-0.2811	O	-3.3085	-2.6161	-1.0411
C	-5.2567	2.887	-0.6849	C	2.0415	-2.6415	1.9179
C	6.6024	-2.1254	-0.2563	C	-2.0741	-2.926	-1.6796
O	-1.7407	0.7578	2.3119	O	2.1268	2.649	1.4418
O	1.0024	-0.1746	-1.9919	O	-1.9291	2.5683	-1.6676
H	-0.7988	0.7435	-0.1803	H	0.6588	1.7099	-1.4641
H	-5.5203	-2.8119	0.0463	H	5.6175	-0.199	-1.8667
H	-3.3062	1.6236	0.3947	H	1.7993	-0.1707	1.3111
H	-3.2914	-2.6217	1.1011	H	4.2712	1.8533	-1.5768
H	-7.1476	0.0535	-1.2127	H	4.839	-3.1214	-0.0487
H	-1.5582	-1.2421	2.0484	H	2.5803	3.0472	-0.493
H	-1.1878	-1.3204	-1.4129	H	0.0979	4.0414	-1.2496
H	-0.6068	-2.3186	-0.0521	H	0.1042	3.9716	0.5299
H	1.401	-2.1065	-1.4244	H	-2.2583	3.6706	0.0342
H	1.7702	-1.7021	0.9498	H	-1.362	2.0709	1.6533
H	4.0664	-1.8677	0.0242	H	-1.368	-0.6647	-0.7196
H	4.2177	3.0998	0.1105	H	-5.74	0.1787	1.4871
H	2.0561	1.9254	0.372	H	-3.9113	1.8319	1.6976
H	7.206	1.2702	-0.3201	H	-5.2629	-2.7868	-0.3636
H	-5.9701	3.5921	-1.1223	H	1.9914	-3.6623	2.3087
H	-5.1231	3.1587	0.3675	H	2.1284	-1.9622	2.7722
H	-4.3176	2.9762	-1.2408	H	1.1213	-2.4555	1.3537
H	7.6454	-2.4311	-0.3831	H	-2.1879	-3.8973	-2.1707

H	6.2557	-2.5144	0.7069	H	-1.8339	-2.1885	-2.4525
H	6.0274	-2.5503	-1.0856	H	-1.2629	-3.0154	-0.9493
H	-0.7942	0.901	2.5079	H	2.9047	2.2371	1.8559
H	1.9327	0.085	-2.1218	H	-2.774	2.0851	-1.6161
**2-7**	X axis(Å)	Y axis(Å)	Z axis(Å)	**2-8**	X axis(Å)	Y axis(Å)	Z axis(Å)
C	5.0606	-1.1362	-1.1923	C	-5.0169	1.1525	-1.3706
C	5.6395	-0.4185	-0.1548	C	-5.6475	0.7669	-0.1957
C	4.9319	0.5684	0.5389	C	-5.0642	-0.1517	0.6827
C	3.6086	0.8244	0.1777	C	-3.8118	-0.6799	0.367
C	2.9979	0.1081	-0.8711	C	-3.1501	-0.3026	-0.8187
C	3.7399	-0.8718	-1.5514	C	-3.7687	0.6161	-1.683
O	6.9372	-0.6983	0.1788	O	-6.8718	1.3092	0.0882
O	5.6367	1.1951	1.5345	O	-5.8078	-0.4395	1.7985
C	1.5552	0.3588	-1.247	C	-1.779	-0.8468	-1.1507
C	0.5543	-0.2802	-0.2668	C	-0.6511	-0.1691	-0.3509
C	0.5992	-1.8004	-0.2067	C	-0.4757	1.3182	-0.6234
C	-0.8194	-2.1288	0.1982	C	0.9982	1.5083	-0.3478
C	-1.6132	-1.1172	-0.6494	C	1.5874	0.2295	-0.9693
O	-0.794	0.0781	-0.6757	O	0.6106	-0.804	-0.6913
C	-3.0286	-0.8014	-0.2084	C	2.9698	-0.2013	-0.5212
C	-3.5702	0.4859	-0.4096	C	3.9368	0.7363	-0.1046
C	-4.8839	0.7797	-0.0379	C	5.2171	0.3258	0.2762
C	-5.665	-0.2321	0.5264	C	5.5369	-1.0332	0.213
C	-5.1653	-1.5114	0.7192	C	4.6131	-1.9756	-0.212
C	-3.852	-1.7968	0.3459	C	3.335	-1.5586	-0.5821
O	-6.9567	0.0209	0.9003	O	6.7844	-1.4658	0.5723
O	-5.5143	1.9898	-0.1739	O	6.232	1.1389	0.7124
C	4.9711	2.2554	2.2114	C	-5.2862	-1.4394	2.6667
C	-4.744	3.0521	-0.7245	C	5.9123	2.5127	0.902
O	1.3199	1.7738	-1.2783	O	-1.7546	-2.253	-0.8694
O	-0.9313	-1.9217	1.6093	O	1.1797	1.5964	1.0688
H	0.6821	0.1491	0.7353	H	-0.7914	-0.3493	0.7228
H	5.6364	-1.8928	-1.7167	H	-5.4972	1.8626	-2.037
H	3.0233	1.5846	0.6865	H	-3.3234	-1.3984	1.0187
H	3.2954	-1.4363	-2.368	H	-3.2837	0.9203	-2.6079
H	7.1431	-0.0852	0.9112	H	-7.134	0.9001	0.9359
H	1.359	0.0058	-2.2666	H	-1.5826	-0.7582	-2.2261
H	1.3546	-2.1567	0.5	H	-1.1263	1.9267	0.012
H	0.8087	-2.2235	-1.1963	H	-0.6873	1.5491	-1.6741
H	-1.0909	-3.1671	-0.012	H	1.3983	2.4227	-0.795
H	-1.6596	-1.4736	-1.6871	H	1.6112	0.34	-2.0618
H	-2.9407	1.2455	-0.8629	H	3.6719	1.7884	-0.0795
H	-5.7952	-2.2811	1.1555	H	4.8863	-3.0255	-0.2568

H	-3.4821	-2.8085	0.5008	H	2.6198	-2.3058	-0.9214
H	-7.1021	0.9613	0.6792	H	7.2655	-0.6561	0.8316
H	5.6678	2.6713	2.9457	H	-6.0053	-1.5838	3.4788
H	4.6991	3.0586	1.5185	H	-5.1744	-2.3968	2.1469
H	4.0954	1.8866	2.7555	H	-4.34	-1.1173	3.1139
H	-5.3781	3.9435	-0.7537	H	6.8007	3.0094	1.3037
H	-3.8779	3.2801	-0.0944	H	5.6645	2.9929	-0.0502
H	-4.4394	2.8266	-1.7518	H	5.1041	2.6351	1.6308
H	0.3496	1.8672	-1.3299	H	-0.812	-2.5051	-0.9116
H	-1.319	-1.0399	1.7624	H	1.3084	0.6928	1.415

Table S3. The Cartesian coordinates of the lowest energy conformers for **3**

**3-1**	X axis(Å)	Y axis(Å)	Z axis(Å)	**3-2**	X axis(Å)	Y axis(Å)	Z axis(Å)
C	-5.0432	-0.1349	1.2017	C	5.4354	0.3673	-0.7195
C	-4.8954	-1.1674	0.2856	C	5.2674	-0.8843	-0.1433
C	-3.7808	-1.2479	-0.5543	C	4.0446	-1.2787	0.4074
C	-2.8028	-0.2568	-0.4671	C	2.9765	-0.3812	0.3818
C	-2.9264	0.7986	0.459	C	3.1187	0.8922	-0.2049
C	-4.0581	0.8479	1.2893	C	4.3609	1.2553	-0.7513
O	-5.8764	-2.1195	0.2218	O	6.3376	-1.7369	-0.1269
O	-3.7699	-2.3236	-1.4044	O	4.0266	-2.5429	0.938
C	-1.8639	1.8792	0.5572	C	1.9616	1.8756	-0.2321
C	-0.4974	1.3568	1.081	C	0.7438	1.3882	-1.0662
C	0.5011	2.4675	1.3833	C	-0.3124	2.4664	-1.2779
C	1.2717	2.6327	0.094	C	-1.2646	2.2839	-0.1188
C	1.1918	1.2449	-0.5593	C	-1.1282	0.796	0.2357
O	0.1473	0.5072	0.1114	O	0.0602	0.2993	-0.4147
C	2.5065	0.4883	-0.493	C	-2.3446	-0.0143	-0.1739
C	2.7107	-0.5849	0.3965	C	-3.5198	0.0791	0.6014
C	3.9355	-1.2542	0.4367	C	-4.665	-0.6372	0.2501
C	4.9604	-0.8372	-0.4187	C	-4.6266	-1.452	-0.8856
C	4.7852	0.2168	-1.3038	C	-3.4832	-1.5605	-1.6645
C	3.5587	0.8779	-1.3414	C	-2.3431	-0.8407	-1.3091
O	6.1733	-1.4712	-0.4002	O	-5.7325	-2.1679	-1.2573
O	4.256	-2.3096	1.2521	O	-5.8632	-0.6291	0.9171
C	-2.6032	-2.4889	-2.2037	C	2.7624	-3.0261	1.3808
C	3.2214	-2.7949	2.1002	C	-5.9466	0.2048	2.0673
O	-1.7248	2.534	-0.7115	O	1.5793	2.2051	1.1112
O	0.6628	3.6389	-0.7189	O	-0.885	3.1252	0.9733
H	-0.6718	0.7612	1.9852	H	1.1036	1.0271	-2.037
H	-5.9198	-0.0989	1.842	H	6.3967	0.6461	-1.141
H	-1.9265	-0.2795	-1.1089	H	2.016	-0.6472	0.8135

H	-4.1853	1.6521	2.0113	H	4.5055	2.2335	-1.2055
H	-5.5791	-2.7393	-0.4727	H	6.0063	-2.546	0.3097
H	-2.2199	2.6485	1.2534	H	2.3192	2.8112	-0.6793
H	1.189	2.1119	2.1606	H	-0.8479	2.247	-2.21
H	0.0253	3.3851	1.7414	H	0.111	3.472	-1.3554
H	2.2955	2.9722	0.2788	H	-2.284	2.5788	-0.3868
H	0.8916	1.3171	-1.612	H	-0.9711	0.6564	1.3124
H	1.8942	-0.8824	1.0465	H	-3.5086	0.7243	1.4753
H	5.5971	0.5196	-1.9581	H	-3.4798	-2.2017	-2.5407
H	3.4263	1.7048	-2.0374	H	-1.4508	-0.9351	-1.9244
H	6.0801	-2.1676	0.2786	H	-6.4076	-1.9511	-0.5853
H	-2.7258	-3.4072	-2.7861	H	2.8982	-4.0606	1.7106
H	-2.4901	-1.6597	-2.9097	H	2.4002	-2.4487	2.2376
H	-1.7096	-2.6059	-1.5815	H	2.0306	-3.0296	0.5659
H	3.6226	-3.6426	2.664	H	-6.9567	0.1078	2.4769
H	2.9132	-2.0314	2.8221	H	-5.2415	-0.1181	2.8403
H	2.3686	-3.1572	1.5165	H	-5.7935	1.2567	1.8044
H	-2.5745	2.4252	-1.179	H	2.3513	2.0167	1.6778
H	-0.2552	3.3442	-0.9115	H	0.0269	2.8674	1.2366
**3-3**	X axis(Å)	Y axis(Å)	Z axis(Å)	**3-4**	X axis(Å)	Y axis(Å)	Z axis(Å)
C	-5.0071	-1.6944	0.1017	C	-4.9681	-1.5217	-0.1037
C	-5.5536	-0.6012	-0.5564	C	-5.444	-0.2818	-0.507
C	-4.933	0.6519	-0.5376	C	-4.7148	0.8907	-0.2857
C	-3.7275	0.7936	0.1506	C	-3.4731	0.8005	0.3446
C	-3.1511	-0.3017	0.8243	C	-2.9677	-0.4466	0.7634
C	-3.8067	-1.544	0.7942	C	-3.731	-1.6037	0.5337
O	-6.7318	-0.776	-1.2307	O	-6.6611	-0.2285	-1.1309
O	-5.5934	1.6368	-1.2265	O	-5.3146	2.04	-0.7334
C	-1.8261	-0.1657	1.5414	C	-1.6092	-0.5588	1.4196
C	-0.6232	-0.2214	0.5841	C	-0.4545	-0.5865	0.4033
C	-0.4401	-1.5411	-0.1377	C	-0.4236	-1.805	-0.4999
C	1.0253	-1.4855	-0.4995	C	1.0356	-1.8576	-0.886
C	1.6398	-0.8127	0.7392	C	1.7144	-1.4727	0.4348
O	0.5916	-0.0226	1.347	O	0.8049	-0.5888	1.1262
C	2.8546	0.0307	0.401	C	3.0612	-0.8152	0.2224
C	4.044	-0.6131	-4E-4	C	3.1807	0.5774	0.0443
C	5.1802	0.1264	-0.332	C	4.4286	1.161	-0.1801
C	5.1192	1.5212	-0.2583	C	5.5574	0.3372	-0.2353
C	3.9625	2.1785	0.1365	C	5.465	-1.0369	-0.064
C	2.8309	1.4332	0.4663	C	4.2168	-1.613	0.1652
O	6.215	2.2764	-0.5782	O	6.7945	0.8775	-0.4604
O	6.3881	-0.3807	-0.7375	O	4.6773	2.4968	-0.365
C	-5.0559	2.9508	-1.1228	C	-4.6619	3.2623	-0.4084

C	6.4896	-1.7983	-0.8127	C	3.5664	3.376	-0.2296
O	-1.7936	1.0888	2.2359	O	-1.4111	0.5633	2.2925
O	1.5446	-2.7875	-0.7347	O	1.3863	-3.1521	-1.3484
H	-0.6689	0.6079	-0.1343	H	-0.4526	0.3341	-0.1952
H	-5.5158	-2.6534	0.0772	H	-5.5585	-2.4153	-0.283
H	-3.2114	1.7485	0.1915	H	-2.8736	1.6856	0.5352
H	-3.387	-2.4042	1.3114	H	-3.3667	-2.5793	0.8489
H	-6.9429	0.1056	-1.5954	H	-6.8061	0.7204	-1.313
H	-1.7308	-0.9358	2.3164	H	-1.5702	-1.4449	2.0646
H	-1.0869	-1.6488	-1.0136	H	-1.0878	-1.7178	-1.3651
H	-0.6333	-2.3953	0.5232	H	-0.7048	-2.7182	0.0396
H	1.175	-0.879	-1.4016	H	1.235	-1.1298	-1.6825
H	1.9387	-1.5598	1.4854	H	1.8435	-2.3523	1.0784
H	4.0485	-1.6989	-0.0462	H	2.2814	1.1836	0.0893
H	3.9434	3.2629	0.1889	H	6.3596	-1.6512	-0.1057
H	1.9313	1.9593	0.7778	H	4.1528	-2.6906	0.3051
H	6.905	1.6267	-0.815	H	6.6379	1.8386	-0.5403
H	-5.7184	3.6261	-1.6729	H	-5.286	4.0818	-0.7777
H	-5.028	3.2872	-0.081	H	-4.5612	3.3828	0.6754
H	-4.0651	3.0073	-1.5853	H	-3.6902	3.3334	-0.908
H	7.5042	-2.0428	-1.1416	H	3.9321	4.3986	-0.3636
H	6.3384	-2.2577	0.1698	H	2.8179	3.1859	-1.0058
H	5.7927	-2.205	-1.5529	H	3.1268	3.3078	0.771
H	-0.87	1.1826	2.5412	H	-0.472	0.5098	2.5576
H	1.0801	-3.1501	-1.5098	H	2.2997	-3.1003	-1.6835
**3-5**	X axis(Å)	Y axis(Å)	Z axis(Å)	**3-6**	X axis(Å)	Y axis(Å)	Z axis(Å)
C	-4.9681	-1.5217	-0.1037	C	3.2889	2.145	-0.4988
C	-5.444	-0.2818	-0.507	C	4.6296	1.7928	-0.4424
C	-4.7148	0.8907	-0.2857	C	5.032	0.5077	-0.0708
C	-3.4731	0.8005	0.3446	C	4.0532	-0.4319	0.257
C	-2.9677	-0.4466	0.7634	C	2.6812	-0.096	0.2238
C	-3.731	-1.6037	0.5337	C	2.3157	1.2	-0.1716
O	-6.6611	-0.2285	-1.1309	O	5.5611	2.7402	-0.7695
O	-5.3146	2.04	-0.7334	O	6.3874	0.302	-0.0708
C	-1.6092	-0.5588	1.4196	C	1.6693	-1.1759	0.573
C	-0.4545	-0.5865	0.4033	C	0.2597	-0.697	1.0172
C	-0.4236	-1.805	-0.4999	C	-0.5799	-1.7977	1.6569
C	1.0356	-1.8576	-0.886	C	-1.3521	-2.3921	0.5036
C	1.7144	-1.4727	0.4348	C	-1.4924	-1.2188	-0.4774
O	0.8049	-0.5888	1.1262	O	-0.4969	-0.233	-0.1204
C	3.0612	-0.8152	0.2224	C	-2.8824	-0.6068	-0.4655
C	3.1807	0.5774	0.0443	C	-3.1491	0.6419	0.1298
C	4.4286	1.161	-0.1801	C	-4.4419	1.1701	0.1296

C	5.5574	0.3372	-0.2353	C	-5.471	0.4352	-0.4675
C	5.465	-1.0369	-0.064	C	-5.2348	-0.7958	-1.0621
C	4.2168	-1.613	0.1652	C	-3.9414	-1.3149	-1.0625
O	6.7945	0.8775	-0.4604	O	-6.7493	0.9243	-0.4795
O	4.6773	2.4968	-0.365	O	-4.8275	2.3695	0.6723
C	-4.6619	3.2623	-0.4084	C	6.8351	-1.0044	0.2728
C	3.5664	3.376	-0.2296	C	-3.796	3.178	1.2265
O	-1.4111	0.5633	2.2925	O	1.5542	-2.0596	-0.5519
O	1.3863	-3.1521	-1.3484	O	-0.6256	-3.4836	-0.0664
H	-0.4526	0.3341	-0.1952	H	0.3668	0.1338	1.7247
H	-5.5585	-2.4153	-0.283	H	3.0032	3.1482	-0.8016
H	-2.8736	1.6856	0.5352	H	4.3256	-1.4451	0.5399
H	-3.3667	-2.5793	0.8489	H	1.2724	1.4989	-0.2403
H	-6.8061	0.7204	-1.313	H	6.4226	2.2899	-0.6724
H	-1.5702	-1.4449	2.0646	H	2.0911	-1.7589	1.4016
H	-1.0878	-1.7178	-1.3651	H	-1.2889	-1.3318	2.3527
H	-0.7048	-2.7182	0.0396	H	0.0183	-2.5233	2.2158
H	1.235	-1.1298	-1.6825	H	-2.3106	-2.8061	0.8315
H	1.8435	-2.3523	1.0784	H	-1.2642	-1.5295	-1.5043
H	2.2814	1.1836	0.0893	H	-2.3285	1.1864	0.5851
H	6.3596	-1.6512	-0.1057	H	-6.0514	-1.3446	-1.5215
H	4.1528	-2.6906	0.3051	H	-3.7617	-2.2812	-1.5315
H	6.6379	1.8386	-0.5403	H	-6.6907	1.7865	-0.0238
H	-5.286	4.0818	-0.7777	H	7.9279	-1.008	0.2165
H	-4.5612	3.3828	0.6754	H	6.5537	-1.2601	1.2996
H	-3.6902	3.3334	-0.908	H	6.4624	-1.7491	-0.4382
H	3.9321	4.3986	-0.3636	H	-4.2548	4.1045	1.585
H	2.8179	3.1859	-1.0058	H	-3.3278	2.685	2.0848
H	3.1268	3.3078	0.771	H	-3.0527	3.4463	0.4684
H	-0.472	0.5098	2.5576	H	1.6633	-1.4985	-1.3449
H	2.2997	-3.1003	-1.6835	H	0.2446	-3.143	-0.3701
**3-7**	X axis(Å)	Y axis(Å)	Z axis(Å)	**3-8**	X axis(Å)	Y axis(Å)	Z axis(Å)
C	-3.3083	-1.4572	-1.5498	C	-3.7082	-2.1478	-0.3247
C	-4.5339	-1.5318	-0.9039	C	-4.992	-1.6342	-0.4375
C	-4.87	-0.6557	0.1329	C	-5.2587	-0.2805	-0.2179
C	-3.9431	0.3135	0.5228	C	-4.2016	0.5624	0.1292
C	-2.6892	0.4061	-0.1151	C	-2.8865	0.0635	0.2652
C	-2.3878	-0.4873	-1.1569	C	-2.6559	-1.299	0.0211
O	-5.4202	-2.4924	-1.3094	O	-6.0033	-2.4901	-0.779
O	-6.1161	-0.8494	0.6714	O	-6.5706	0.0848	-0.3748
C	-1.6976	1.4754	0.3127	C	-1.7816	1.0441	0.6252
C	-0.3729	0.9047	0.8915	C	-0.4758	0.4462	1.2169
C	0.4986	1.9597	1.562	C	0.4405	1.4955	1.8385

C	1.4039	2.4471	0.4551	C	1.3643	1.8952	0.713
C	1.5126	1.2368	-0.4854	C	1.4297	0.6365	-0.1631
O	0.4453	0.3257	-0.1432	O	0.2988	-0.1935	0.1811
C	2.8607	0.5424	-0.3958	C	2.731	-0.1259	0.0106
C	3.0145	-0.7247	0.2004	C	3.8986	0.3671	-0.6098
C	4.2697	-1.3325	0.2699	C	5.1195	-0.2928	-0.46
C	5.3758	-0.6588	-0.2581	C	5.1652	-1.454	0.3177
C	5.2513	0.5881	-0.8534	C	4.0309	-1.9593	0.9374
C	3.9944	1.1863	-0.9246	C	2.8147	-1.2948	0.7843
O	6.6199	-1.2267	-0.2001	O	6.3466	-2.1243	0.4858
O	4.5474	-2.5577	0.8211	O	6.3186	0.0854	-1.0072
C	-6.5037	0.0272	1.7228	C	-6.878	1.4605	-0.18
C	3.4343	-3.3086	1.2928	C	6.3118	1.2631	-1.8059
O	-1.4472	2.3745	-0.7766	O	-1.4767	1.8189	-0.5444
O	0.8204	3.5788	-0.1953	O	0.8262	3.0127	0.0012
H	-0.6143	0.1148	1.6128	H	-0.732	-0.3051	1.9733
H	-3.0716	-2.1495	-2.3525	H	-3.5266	-3.2027	-0.5098
H	-4.1741	1.0112	1.3217	H	-4.3663	1.6232	0.2969
H	-1.429	-0.4354	-1.6704	H	-1.658	-1.7266	0.0876
H	-6.2021	-2.3727	-0.7363	H	-6.8055	-1.9333	-0.8069
H	-2.1691	2.081	1.0963	H	-2.1929	1.7338	1.3733
H	1.112	1.4669	2.3266	H	1.033	1.0139	2.6264
H	-0.0818	2.7503	2.0465	H	-0.1057	2.3307	2.2866
H	2.3673	2.7878	0.8467	H	2.3405	2.2183	1.0875
H	1.3486	1.5281	-1.5301	H	1.316	0.8851	-1.2253
H	2.1358	-1.22	0.6006	H	3.8217	1.2734	-1.2037
H	6.1255	1.0878	-1.2596	H	4.0938	-2.8648	1.5332
H	3.9017	2.1639	-1.3952	H	1.9313	-1.7023	1.2708
H	6.4802	-2.0865	0.2421	H	6.9999	-1.612	-0.0291
H	-7.5136	-0.2541	2.0367	H	-7.952	1.589	-0.3458
H	-5.8433	-0.0806	2.5896	H	-6.66	1.7717	0.8469
H	-6.5392	1.0657	1.3777	H	-6.3515	2.0892	-0.9057
H	3.8116	-4.2664	1.6637	H	7.3319	1.4277	-2.1661
H	2.9418	-2.7995	2.1278	H	5.665	1.1426	-2.6813
H	2.7274	-3.5202	0.4837	H	6.0218	2.1385	-1.2155
H	-2.1291	2.2044	-1.4533	H	-1.5814	1.2082	-1.3008
H	-0.0354	3.2869	-0.5817	H	-0.0623	2.7578	-0.3329
**3-9**	X axis(Å)	Y axis(Å)	Z axis(Å)	**3-10**	X axis(Å)	Y axis(Å)	Z axis(Å)
C	-3.5905	-1.7289	-1.2507	C	-4.6493	-1.62	-0.6137
C	-4.8715	-1.5345	-0.7548	C	-5.3218	-0.414	-0.7552
C	-5.1862	-0.4348	0.0496	C	-4.8024	0.7804	-0.2451
C	-4.1807	0.4848	0.3563	C	-3.5686	0.7523	0.4059

C	-2.87	0.3065	-0.1317	C	-2.8634	-0.4582	0.5556
C	-2.5914	-0.8076	-0.9412	C	-3.4215	-1.6423	0.0456
O	-5.8345	-2.4524	-1.0751	O	-6.5248	-0.418	-1.4083
O	-6.4928	-0.3755	0.4612	O	-5.5838	1.8883	-0.4527
C	-1.794	1.3282	0.1971	C	-1.5112	-0.495	1.2314
C	-0.5892	0.7463	0.9885	C	-0.3752	0.1223	0.3792
C	0.3411	1.8191	1.5429	C	-0.0901	-0.5513	-0.9538
C	1.3757	2.0067	0.4578	C	0.8368	-1.6814	-0.5757
C	1.4147	0.6428	-0.2471	C	1.5969	-1.1273	0.6427
O	0.2359	-0.0875	0.1522	O	0.8551	0.0139	1.136
C	2.6724	-0.1423	0.0797	C	3.0215	-0.7312	0.3023
C	3.8872	0.2276	-0.5353	C	3.3887	0.607	0.0594
C	5.0696	-0.4562	-0.2479	C	4.7045	0.9353	-0.2727
C	5.0289	-1.5169	0.6623	C	5.6514	-0.0893	-0.3693
C	3.8466	-1.9006	1.2794	C	5.3152	-1.415	-0.1348
C	2.669	-1.2128	0.9884	C	4.0013	-1.7352	0.2018
O	6.1711	-2.2071	0.967	O	6.9478	0.1996	-0.6994
O	6.3078	-0.1924	-0.7751	O	5.1856	2.1938	-0.5293
C	-6.8552	0.7237	1.2887	C	-5.1539	3.0915	0.1745
C	6.3882	0.8806	-1.7064	C	4.273	3.2715	-0.3534
O	-1.3614	1.9852	-1.003	O	-1.599	0.2531	2.456
O	0.9778	3.0538	-0.4309	O	0.0586	-2.8246	-0.2109
H	-0.9712	0.1289	1.8103	H	-0.5663	1.1925	0.2366
H	-3.3714	-2.592	-1.8726	H	-5.0822	-2.5338	-1.0089
H	-4.3934	1.3521	0.9735	H	-3.1294	1.6543	0.8216
H	-1.59	-0.9688	-1.337	H	-2.9008	-2.5907	0.1618
H	-6.6436	-2.1355	-0.629	H	-6.8286	0.5101	-1.3751
H	-2.2491	2.1103	0.8171	H	-1.2644	-1.5167	1.5387
H	0.837	1.4187	2.436	H	0.4554	0.1538	-1.5932
H	-0.1826	2.737	1.8254	H	-0.9889	-0.8684	-1.4879
H	2.341	2.3137	0.8723	H	1.4907	-1.9863	-1.3992
H	1.3538	0.7555	-1.3365	H	1.621	-1.8562	1.462
H	3.8765	1.0587	-1.2347	H	2.6277	1.3765	0.14
H	3.8418	-2.7305	1.9795	H	6.0717	-2.1901	-0.2121
H	1.7464	-1.5246	1.4734	H	3.7438	-2.7767	0.3869
H	6.8682	-1.7854	0.428	H	6.9675	1.1704	-0.8081
H	-7.9172	0.6226	1.5319	H	-5.9051	3.8606	-0.0297
H	-6.2952	0.7108	2.2296	H	-5.0872	2.9714	1.261
H	-6.7231	1.6743	0.7614	H	-4.2033	3.4354	-0.2462
H	7.4297	0.962	-2.0323	H	4.8133	4.2019	-0.5534
H	5.7768	0.6808	-2.5925	H	3.446	3.2069	-1.0681
H	6.1107	1.8308	-1.2384	H	3.9051	3.3151	0.6771
H	-1.9873	1.7357	-1.7086	H	-0.6796	0.3102	2.7805

H	0.1188	2.7884	-0.8297	H	-0.3111	-3.1958	-1.0326
**3-11**	X axis(Å)	Y axis(Å)	Z axis(Å)	**3-12**	X axis(Å)	Y axis(Å)	Z axis(Å)
C	-4.604	-1.5254	-0.8735	C	-5.0017	-1.7644	-0.0036
C	-5.2989	-0.3241	-0.8585	C	-5.6408	-0.6139	-0.4444
C	-4.8094	0.7982	-0.182	C	-5.0666	0.6512	-0.2844
C	-3.5849	0.7023	0.4804	C	-3.8142	0.7479	0.3233
C	-2.8601	-0.5059	0.4791	C	-3.1446	-0.4054	0.7791
C	-3.3859	-1.6166	-0.2021	C	-3.7544	-1.6598	0.6101
O	-6.4926	-0.2588	-1.5256	O	-6.8643	-0.7437	-1.0443
O	-5.6086	1.911	-0.2503	O	-5.8174	1.6939	-0.764
C	-1.5201	-0.6163	1.1721	C	-1.7729	-0.3128	1.4101
C	-0.354	0.0051	0.3653	C	-0.6429	-0.2089	0.3712
C	-0.024	-0.6604	-0.961	C	-0.4648	-1.4336	-0.5073
C	0.9099	-1.7776	-0.5612	C	0.9815	-1.2988	-0.9211
C	1.6442	-1.2013	0.6609	C	1.623	-0.8002	0.3795
O	0.8497	-0.1055	1.1689	O	0.6178	-0.0265	1.0685
C	3.0484	-0.7357	0.3265	C	2.87	0.0209	0.1339
C	3.337	0.6126	0.0364	C	4.134	-0.6012	0.183
C	4.6348	1.0092	-0.2916	C	5.296	0.1292	-0.0719
C	5.6441	0.0425	-0.3346	C	5.1827	1.4875	-0.3856
C	5.386	-1.2916	-0.0534	C	3.9492	2.1212	-0.4405
C	4.089	-1.6803	0.2775	C	2.7929	1.3877	-0.1808
O	6.9255	0.3978	-0.6578	O	6.3021	2.2301	-0.647
O	5.0416	2.2841	-0.591	O	6.5783	-0.3558	-0.0551
C	-5.2028	3.0292	0.5311	C	-5.3166	3.0023	-0.5126
C	4.0572	3.3069	-0.4898	C	6.7439	-1.7125	0.3407
O	-1.5988	0.075	2.4322	O	-1.7188	0.8475	2.2528
O	0.1241	-2.9189	-0.2053	O	1.4998	-2.5433	-1.3638
H	-0.5409	1.0762	0.2231	H	-0.7748	0.6897	-0.246
H	-5.0089	-2.3827	-1.4024	H	-5.4739	-2.7332	-0.1358
H	-3.1679	1.5476	1.0195	H	-3.3318	1.7101	0.4678
H	-2.8419	-2.5597	-0.218	H	-3.2612	-2.5662	0.955
H	-6.8108	0.6525	-1.375	H	-7.1336	0.1705	-1.2595
H	-1.3121	-1.6583	1.4354	H	-1.6004	-1.1681	2.0746
H	0.5253	0.0546	-1.5859	H	-1.1494	-1.4557	-1.3606
H	-0.9026	-0.9913	-1.5192	H	-0.6116	-2.3635	0.0564
H	1.5676	-2.0798	-1.3826	H	1.0669	-0.5683	-1.7353
H	1.702	-1.9379	1.4715	H	1.8707	-1.643	1.0375
H	2.5292	1.3364	0.0774	H	4.1814	-1.6586	0.4256
H	6.1908	-2.0198	-0.0905	H	3.8906	3.1786	-0.681
H	3.8976	-2.728	0.4997	H	1.8293	1.8914	-0.2205
H	6.8878	1.3628	-0.8068	H	7.0475	1.6071	-0.545

H	-5.9631	3.8081	0.4182	H	-6.045	3.7189	-0.9043
H	-5.1458	2.7713	1.5939	H	-5.212	3.1877	0.5617
H	-4.2531	3.4369	0.1697	H	-4.3708	3.1686	-1.0384
H	4.5408	4.2597	-0.7258	H	7.816	-1.9318	0.3405
H	3.2534	3.1526	-1.2172	H	6.3703	-1.8769	1.3569
H	3.6626	3.3771	0.5293	H	6.2649	-2.3911	-0.3726
H	-0.6747	0.1314	2.7435	H	-0.7733	0.9502	2.4781
H	0.7369	-3.6602	-0.0515	H	2.3745	-2.3653	-1.7543

Table S4. The Cartesian coordinates of the lowest energy conformers for **4**

**4-1**	X axis(Å)	Y axis(Å)	Z axis(Å)	**4-2**	X axis(Å)	Y axis(Å)	Z axis(Å)
C	4.7446	1.2246	-1.6139	C	5.0913	-1.4154	-0.2341
C	5.5153	0.6082	-0.6382	C	5.4109	-0.065	-0.2377
C	4.936	0.0198	0.4905	C	4.4272	0.9207	-0.3623
C	3.5472	0.0484	0.6252	C	3.0935	0.5274	-0.4838
C	2.7412	0.6662	-0.3514	C	2.7412	-0.837	-0.4795
C	3.3585	1.255	-1.4694	C	3.7571	-1.801	-0.355
O	6.8741	0.5901	-0.8031	O	6.7277	0.2877	-0.1151
O	5.8239	-0.5465	1.3695	O	4.8939	2.2103	-0.3477
C	1.2263	0.6849	-0.2376	C	1.2993	-1.287	-0.5871
C	0.5702	-0.4031	-1.1296	C	0.701	-1.791	0.7496
C	0.5527	-1.8203	-0.5492	C	0.7086	-0.8119	1.9213
C	-0.8703	-1.9828	-0.0625	C	-0.6254	-0.1124	1.8215
C	-1.639	-1.1795	-1.116	C	-1.5354	-1.2298	1.2736
O	-0.8106	-0.0334	-1.3824	O	-0.6861	-2.114	0.5004
C	-3.0366	-0.7878	-0.6919	C	-2.7564	-0.835	0.4648
C	-3.316	0.4908	-0.1696	C	-3.4244	0.3866	0.682
C	-4.6047	0.8229	0.2499	C	-4.5633	0.7241	-0.0538
C	-5.6147	-0.1384	0.1447	C	-5.0543	-0.1865	-0.9932
C	-5.3646	-1.4047	-0.3661	C	-4.434	-1.407	-1.2113
C	-4.0751	-1.73	-0.7835	C	-3.2917	-1.7307	-0.48
O	-6.8885	0.1536	0.5509	O	-6.1726	0.1114	-1.7235
O	-5.0009	2.025	0.777	O	-5.2821	1.8874	0.0555
C	5.2753	-1.062	2.5777	C	3.919	3.2392	-0.4808
C	-4.0016	3.0341	0.8798	C	-4.7328	2.8962	0.8963
O	0.8464	0.6031	1.138	O	0.4666	-0.2491	-1.1279
O	-0.9484	-1.3921	1.2418	O	-0.4886	1.0372	0.9862
H	1.063	-0.4163	-2.1093	H	1.2168	-2.7132	1.0423
H	5.2213	1.6792	-2.477	H	5.8767	-2.1593	-0.1398
H	3.0587	-0.3987	1.486	H	2.3025	1.2652	-0.5797
H	2.7598	1.7436	-2.2358	H	3.5167	-2.8623	-0.3558
H	7.2135	0.1302	-0.0106	H	6.7275	1.2643	-0.15

H	0.8728	1.669	-0.5704	H	1.2543	-2.0999	-1.3241
H	0.7448	-2.5363	-1.3579	H	0.7324	-1.3905	2.8535
H	1.3077	-1.979	0.2257	H	1.5665	-0.1357	1.928
H	-1.1869	-3.0272	0.0111	H	-0.9781	0.2478	2.7929
H	-1.6942	-1.7409	-2.0581	H	-1.8862	-1.8344	2.1207
H	-2.5035	1.2083	-0.0988	H	-3.0359	1.0695	1.4309
H	-6.1703	-2.1293	-0.4388	H	-4.8389	-2.1018	-1.9407
H	-3.8887	-2.7241	-1.1847	H	-2.8204	-2.6976	-0.6491
H	-6.8468	1.0804	0.8582	H	-6.4432	0.9977	-1.4141
H	6.1043	-1.4323	3.1885	H	4.4428	4.1996	-0.4557
H	4.7658	-0.2783	3.1483	H	3.3992	3.1688	-1.4421
H	4.6073	-1.9061	2.3773	H	3.2124	3.2237	0.3556
H	-4.472	3.9268	1.3032	H	-5.3767	3.7779	0.823
H	-3.1995	2.7266	1.5588	H	-4.7277	2.5743	1.9427
H	-3.6073	3.3014	-0.1062	H	-3.7304	3.1876	0.565
H	0.0332	0.0602	1.1968	H	-0.349	-0.7111	-1.4132
H	-1.8747	-1.4807	1.5402	H	-0.1817	0.7419	0.1023
**4-3**	X axis(Å)	Y axis(Å)	Z axis(Å)	**4-4**	X axis(Å)	Y axis(Å)	Z axis(Å)
C	4.8381	-1.8398	-0.0304	C	4.7611	-1.0631	1.3344
C	5.3736	-0.5935	-0.3232	C	5.5328	-0.2596	0.5072
C	4.5583	0.5071	-0.604	C	4.9539	0.6962	-0.3341
C	3.1729	0.3385	-0.5868	C	3.5645	0.8327	-0.3413
C	2.6031	-0.9157	-0.2896	C	2.7581	0.0264	0.4886
C	3.4535	-2.0006	-0.0132	C	3.3746	-0.9191	1.3248
O	6.7359	-0.4611	-0.331	O	6.8919	-0.4215	0.5326
O	5.2297	1.6727	-0.8718	O	5.8432	1.4156	-1.0909
C	1.1032	-1.116	-0.2424	C	1.247	0.1717	0.4346
C	0.5304	-1.2209	1.1925	C	0.6363	-0.6545	-0.7292
C	0.7912	-0.0424	2.1283	C	0.3816	-2.1363	-0.4397
C	-0.4129	0.8482	1.9385	C	-1.107	-2.191	-0.1776
C	-1.5333	-0.1798	1.6871	C	-1.6194	-1.1255	-1.1512
O	-0.907	-1.3326	1.0691	O	-0.6411	-0.0711	-1.0984
C	-2.746	0.2517	0.8857	C	-3.0117	-0.6245	-0.8406
C	-3.5009	-0.7081	0.1763	C	-3.2196	0.5481	-0.0872
C	-4.6428	-0.3422	-0.5394	C	-4.5109	0.9765	0.222
C	-5.0403	0.9971	-0.5307	C	-5.5964	0.2195	-0.2292
C	-4.3316	1.9582	0.1742	C	-5.4176	-0.9396	-0.9719
C	-3.1924	1.5837	0.8875	C	-4.1249	-1.3629	-1.2762
O	-6.1547	1.3904	-1.2207	O	-6.8759	0.6103	0.059
O	-5.4456	-1.1802	-1.2701	O	-4.8429	2.0913	0.9481
C	4.4288	2.8149	-1.1565	C	5.298	2.4438	-1.9094
C	-5.0771	-2.5543	-1.2957	C	-3.761	2.8853	1.4238
O	0.415	-0.082	-0.9631	O	0.6921	-0.1304	1.7151

O	-0.1553	1.7562	0.8687	O	-1.3157	-1.8275	1.1935
H	0.9037	-2.1421	1.6554	H	1.2759	-0.5599	-1.6154
H	5.4961	-2.6775	0.1808	H	5.235	-1.7918	1.9847
H	2.5084	1.1712	-0.7978	H	3.0773	1.5597	-0.9837
H	3.0431	-2.9824	0.2137	H	2.7729	-1.5437	1.9826
H	6.8929	0.4752	-0.563	H	7.2337	0.2395	-0.1
H	0.8697	-2.042	-0.7845	H	1.0125	1.2309	0.2694
H	0.7926	-0.4156	3.1604	H	0.6136	-2.718	-1.3405
H	1.7466	0.458	1.9552	H	0.9914	-2.5279	0.3788
H	-0.6217	1.4574	2.8234	H	-1.5406	-3.1816	-0.3429
H	-1.8968	-0.5368	2.6602	H	-1.5962	-1.5085	-2.1801
H	-3.1735	-1.7432	0.2054	H	-2.35	1.1057	0.2491
H	-4.6636	2.9921	0.1701	H	-6.2805	-1.5056	-1.3104
H	-2.6542	2.3533	1.4377	H	-3.9942	-2.272	-1.8597
H	-6.4947	0.5731	-1.6336	H	-6.7759	1.437	0.5705
H	5.1031	3.6558	-1.345	H	6.1292	2.9405	-2.4191
H	3.8286	2.6609	-2.0594	H	4.6381	2.0269	-2.6773
H	3.7991	3.0788	-0.3004	H	4.7794	3.1966	-1.3065
H	-5.8076	-3.084	-1.9147	H	-4.185	3.7292	1.9765
H	-4.0918	-2.6903	-1.7538	H	-3.1296	2.3179	2.1155
H	-5.1121	-2.991	-0.2922	H	-3.1749	3.2921	0.5931
H	-0.4893	-0.4329	-1.0977	H	-0.1665	-0.5817	1.5784
H	0.0217	1.239	0.0548	H	-2.2792	-1.8577	1.3535
**4-5**	X axis(Å)	Y axis(Å)	Z axis(Å)	**4-6**	X axis(Å)	Y axis(Å)	Z axis(Å)
C	3.1916	1.9278	-1.0813	C	4.0528	1.97	-0.7267
C	4.4464	1.5771	-0.604	C	5.1689	1.1797	-0.4931
C	4.6373	0.4447	0.1946	C	5.0575	-0.1953	-0.2646
C	3.5334	-0.3488	0.5122	C	3.7881	-0.7771	-0.2668
C	2.2479	-0.0133	0.0441	C	2.6375	0.0053	-0.4988
C	2.0936	1.1332	-0.7567	C	2.7886	1.3823	-0.731
O	5.5101	2.3728	-0.9349	O	6.3985	1.781	-0.4927
O	5.9329	0.2272	0.5903	O	6.2466	-0.8444	-0.0519
C	1.0276	-0.8628	0.3699	C	1.2732	-0.6525	-0.4753
C	0.5076	-1.6182	-0.8913	C	0.7689	-0.988	0.9495
C	-0.0382	-3.0254	-0.6531	C	0.6029	0.1824	1.9158
C	-1.4484	-2.7367	-0.1983	C	-0.8354	0.6051	1.7363
C	-1.8284	-1.5947	-1.1605	C	-1.5322	-0.7355	1.4299
O	-0.6103	-0.8663	-1.4383	O	-0.5383	-1.5879	0.8084
C	-2.944	-0.7018	-0.6615	C	-2.8018	-0.7212	0.6002
C	-2.7054	0.625	-0.2542	C	-3.6721	0.3869	0.5966
C	-3.7448	1.421	0.2294	C	-4.8508	0.3732	-0.1538
C	-5.0313	0.8784	0.299	C	-5.1758	-0.7759	-0.8795
C	-5.2947	-0.4235	-0.1033	C	-4.3528	-1.8915	-0.8753

C	-4.2518	-1.213	-0.5846	C	-3.1722	-1.8641	-0.1337
O	-6.0721	1.6308	0.7712	O	-6.328	-0.8235	-1.6161
O	-3.6385	2.7182	0.6609	O	-5.7599	1.396	-0.2506
C	6.1545	-0.8899	1.444	C	6.1828	-2.2538	0.1306
C	-2.3437	3.3069	0.5966	C	-5.3823	2.6368	0.3365
O	1.3137	-1.7489	1.4531	O	0.2954	0.1428	-1.1611
O	-1.3814	-2.3444	1.1805	O	-0.9114	1.5894	0.7063
H	1.2858	-1.6569	-1.6625	H	1.4366	-1.7355	1.394
H	3.0721	2.8105	-1.7018	H	4.1657	3.0351	-0.9054
H	3.6417	-1.237	1.1271	H	3.6639	-1.8415	-0.0938
H	1.1119	1.4077	-1.1385	H	1.9186	2.0103	-0.9155
H	6.2778	1.951	-0.5021	H	7.032	1.0575	-0.322
H	0.2625	-0.169	0.7417	H	1.3397	-1.5829	-1.0551
H	-0.0701	-3.5667	-1.6071	H	0.7326	-0.1946	2.9383
H	0.5566	-3.6149	0.0492	H	1.3259	0.9871	1.764
H	-2.1154	-3.6003	-0.2748	H	-1.2462	1.0677	2.6392
H	-2.1325	-2.0174	-2.1272	H	-1.7726	-1.2227	2.3845
H	-1.6955	1.0158	-0.3257	H	-3.4099	1.2605	1.185
H	-6.3059	-0.8156	-0.0444	H	-4.63	-2.7756	-1.4412
H	-4.4698	-2.2301	-0.9042	H	-2.5395	-2.75	-0.1268
H	-5.6779	2.5001	0.9816	H	-6.7471	0.0484	-1.4786
H	7.2209	-0.9163	1.6878	H	7.2056	-2.6201	0.2619
H	5.6012	-0.7863	2.3833	H	5.6193	-2.5085	1.0342
H	5.9061	-1.8284	0.9375	H	5.7625	-2.7482	-0.7515
H	-2.4251	4.3326	0.969	H	-6.165	3.366	0.1061
H	-1.6357	2.7742	1.2401	H	-5.3185	2.551	1.4261
H	-1.983	3.3551	-0.4362	H	-4.4444	3.0094	-0.0887
H	0.4352	-2.0743	1.7462	H	-0.4432	-0.4734	-1.3462
H	-2.2731	-2.0359	1.4346	H	-0.5092	1.2209	-0.1093
**4-7**	X axis(Å)	Y axis(Å)	Z axis(Å)	**4-8**	X axis(Å)	Y axis(Å)	Z axis(Å)
C	-5.002	-1.7718	-0.8879	C	5.0708	-1.7662	-0.2033
C	-5.7444	-0.6664	-0.497	C	5.6079	-0.4873	-0.2525
C	-5.1648	0.3951	0.2053	C	4.7949	0.6462	-0.3483
C	-3.8029	0.3386	0.5052	C	3.4104	0.4772	-0.3975
C	-3.0258	-0.7712	0.1185	C	2.8404	-0.8113	-0.3518
C	-3.6438	-1.8248	-0.5783	C	3.6871	-1.928	-0.251
O	-7.0759	-0.6365	-0.8135	O	6.9692	-0.3567	-0.2025
O	-6.0237	1.4148	0.5283	O	5.4672	1.8406	-0.3829
C	-1.536	-0.8405	0.4102	C	1.3387	-1.016	-0.3868
C	-0.6941	-0.4517	-0.8345	C	0.7042	-1.1129	1.0234
C	-0.4921	1.051	-1.0568	C	0.6099	0.1856	1.8214
C	0.9245	1.2872	-0.5801	C	-0.7924	0.6795	1.5496
C	1.5989	-0.0207	-1.0043	C	-1.5761	-0.6368	1.4108

O	0.6279	-1.0412	-0.7147	O	-0.647	-1.6042	0.8735
C	2.9279	-0.2787	-0.3305	C	-2.8667	-0.6263	0.6139
C	4.0889	0.3529	-0.8217	C	-3.6724	0.5266	0.5272
C	5.3229	0.1564	-0.1985	C	-4.8767	0.5102	-0.1813
C	5.3854	-0.6717	0.9268	C	-5.2906	-0.682	-0.782
C	4.2563	-1.3034	1.4283	C	-4.5297	-1.8377	-0.6968
C	3.0283	-1.1072	0.7991	C	-3.3235	-1.8087	0.0026
O	6.5783	-0.879	1.5646	O	-6.4705	-0.7316	-1.4741
O	6.519	0.71	-0.5761	O	-5.7316	1.5703	-0.3487
C	-5.4924	2.4529	1.3446	C	4.6716	3.0181	-0.466
C	6.515	1.4997	-1.7594	C	-5.2698	2.8348	0.1137
O	-1.2378	-0.053	1.5649	O	0.7161	0.0117	-1.1686
O	0.8793	1.4467	0.8437	O	-0.7806	1.4974	0.3803
H	-1.1401	-0.8974	-1.7321	H	1.2531	-1.8625	1.6062
H	-5.4791	-2.5852	-1.4258	H	5.7267	-2.6286	-0.1277
H	-3.3145	1.1432	1.0468	H	2.7467	1.3346	-0.4668
H	-3.0674	-2.6961	-0.8833	H	3.2742	-2.9345	-0.2101
H	-7.4009	0.2041	-0.4362	H	7.1313	0.6056	-0.2509
H	-1.2911	-1.8714	0.6949	H	1.1241	-1.9498	-0.9229
H	-0.5553	1.2615	-2.1315	H	0.6989	-0.0561	2.8881
H	-1.2401	1.6672	-0.5503	H	1.3899	0.91	1.5778
H	1.3852	2.1794	-1.0139	H	-1.1804	1.2977	2.365
H	1.7433	-0.0383	-2.093	H	-1.8223	-0.9977	2.4187
H	4.0019	0.9921	-1.6947	H	-3.3376	1.4355	1.0165
H	4.3322	-1.945	2.3013	H	-4.8719	-2.7531	-1.1692
H	2.146	-1.6057	1.1977	H	-2.7345	-2.7218	0.0695
H	7.2318	-0.3637	1.0533	H	-6.8335	0.173	-1.4086
H	-6.3032	3.1557	1.5594	H	5.3486	3.8776	-0.479
H	-5.1304	2.0604	2.3008	H	4.0913	3.037	-1.3945
H	-4.7063	3.0037	0.8177	H	4.0225	3.1185	0.4102
H	7.5408	1.8346	-1.9409	H	-6.0207	3.5831	-0.1575
H	6.1979	0.9113	-2.6268	H	-5.1726	2.8417	1.2042
H	5.8907	2.3906	-1.6351	H	-4.329	3.1165	-0.3711
H	-0.3309	0.3023	1.4602	H	1.1104	-0.0553	-2.0583
H	1.8016	1.5224	1.1566	H	-0.4499	0.941	-0.3609
